# Effect of thermo-sonication on the physicochemical properties, bioactive compounds, micronutrients, and antioxidant activity of cranberry juice

**DOI:** 10.1016/j.ultsonch.2026.108098

**Published:** 2026-10-06

**Authors:** Mabel Kyei Kwofie, Valerie Orsat

**Affiliations:** Bioresource Engineering Department, McGill University, 21111 Lakeshore, Sainte-Anne-de-Bellevue, QC H9X 3V9, Canada

## Abstract

Fruits such as cranberry (Vaccinium macrocarpon Aiton), that contain several phenolic substances, are processed commercially as juices, requiring treatments to extend shelf-life. Thermo-sonication, the synergistic application of mild heat and ultrasound, is a promising non-thermal liquid foods processing technology. This study examined the kinetics of change of physicochemical properties, micronutrients, key bioactive compounds, and color attributes of thermo-sonicated cranberry juice at three varying temperatures 35 °C, 45 °C, and 55 °C, over varying treatment times of 1, 3, 5, and 7 min in comparison to ultrasonication treatment alone at same time durations. The total carotenoids, total soluble solids (TSS), pH, CIE (Commission Internationale de l’Éclairage) L*a*b* color coordinates, ascorbic acids, procyanidin A1 and B2 concentrations, total anthocyanin content (TAC), total flavonoid content (TFC), total phenolic content (TPC), FRAP (ferric reducing antioxidant power) antioxidant capacity, and DPPH (2,2-diphenyl-1-picrylhydrazyl) radical scavenging activity were measured. The ultrasonication treatments preserved TPC better than the thermo-sonication treatment, resulting in values of 12.85 ± 0.38 mg GAE/100 mL for the ultrasound-only treatment at 3 min (US-3), while progressively decreased TPC values were recorded for thermo-sonication. Enhanced FRAP values of 0.64 to 1.78 mmol at 7 min (TS35-7), were recorded for thermo-sonication at 35 °C, indicating reducing compounds bioaccessibility. Procyanidin A1 and B2 concentrations which ranged from 10.46 to 12.27 and 14.63–15.90 mg/100 mL, respectively, were statistically stable in all thermo-sonication treatment conditions (p > 0.05), an essential finding from the study, knowing the significance of A-type procyanidins in cranberry bioactivity. Decreased total anthocyanin content (TAC) was observed with all treatments, with the thermo-sonicated samples retaining more anthocyanin than ultrasound alone at 7 min (up to 130.70 mg CGE/L for TS-35 °C); similarly, TS-45 °C treated samples had the most redness a* value up to 17.56, while retaining pH stability across all the treatment conditions. These findings suggest that thermo-sonication treatment conditions significantly impact cranberry juice quality characteristics, with temperature and treatment duration equally governing the level of bioactive compound alteration.

## 1 Introduction

Cranberry juice is regarded as a desirable functional beverage product [1], [2], due to its content in highly bioactive substances, such as anthocyanins and phenolic compounds, which have been associated with many health advantages, including the potential prevention of cardiovascular diseases, gastrointestinal disorders, and some malignancies [3], [4], [5], [6]. The pulps of cranberries contain sugars (fructose, sucrose, xylose, arabinose, and glucose) and pectin. The acid content of ripe cranberries ranges from 2 to 3 % (w/w), the principal organic acid being citric acid, together with several other organic acids such as malic, quinic, and benzoic acids [3], [4]. However, several of these interesting compounds in cranberry and other berries, fruits are intrinsically thermolabile in nature [7], [8]. Thermal pasteurization, while efficient for microbiological safety, frequently leads to the breakdown of these antioxidants, undesired color alterations, and loss of natural sensory properties in some food products such as fruits beverages[9], [10], [7], [11], [12], [12], [13]. To counteract these quality losses with heat processing, novel “green” technique such as thermo-sonication are being investigated in this study[14], [15]. Thermo-sonication utilizes ultrasonication’s mechanical effects together with mild heat treatments[16], [17]. This synergistic technique is based on the phenomenon of acoustic cavitation and mild temperature (35–55 °C), which is a rapid development, expansion, and subsequent collapse of tiny bubbles in liquid medium, while producing extreme pressures with moderate temperatures, efficiently deactivating microorganisms and enzymes such as polyphenol oxidase and releasing bioactive constituents from the fruit's juices cell matrixes; the capacity of ultrasound-assisted processing to enhance the extraction and recovery of proteins, polyphenols and other bioactive compounds from diverse plant matrices has been demonstrated repeatedly[18], [19], [20], [21], [22], [23], [24], [25], [26]. The integrated approach predominantly involves two aspects: firstly, ultrasonic cavitation enhances heat transfer through microturbulence and acoustic streaming that disrupt the thermal boundary layer at the probe surface, thereby improving the efficiency with which thermal energy is delivered to the bulk medium [27]. Secondly, the application of a sublethal temperature causes an alteration of bacterial membrane lipids from the gel phase to the liquid crystalline state [28], ensuing in a heightened membrane fluidity and diminished mechanical strength, thereby enhancing microbial sensitivity to ultrasonic stress. Accordingly, thermo-sonication can potentially attain bacterial inactivation like that of a typical heat treatment within a rapid time duration at a lower temperature (Li et al., 2025; [29], however, its operational prospects is influenced by the optimization of processing parameters such as amplitude, temperature, and exposure time [30], [31]. Excessive sonication can produce free radicals, potentially speeding up the oxidation of phenolic substances[32], [22], [33]. The kinetics of the rate of the cranberry juice’s properties loss is vital for improving the processing parameters to attain optimal bioactive compound retention and microbiological reduction[34]; Li et al., 2025; [35]. Preceding studies on different berry juices have demonstrated that kinetic models may effectively monitor the changing patterns of nutrients and microbial loads during and after treatment[16], [36]. This study aims to identify processing parameters of thermo-sonication that maintain the nutritional integrity and functional attributes of the cranberry juice samples while being effective at inactivating microbial loads for consumer safety.

## 2 Materials and methods

### 2.1 Materials

For this project, frozen cranberries were purchased from Salaberry-de-Valleyfield's Super C supermarket. After being cleaned with distilled water, the cranberries were refrigerated to defrost. Folin-Ciocalteu reagent, sodium acetate trihydrate, glacial acetic acid, hydrogen chloride (HCL), ferric chloride, Trolox, potassium chloride, methanol, sodium nitrate, aluminum chloride, sodium hydroxide, quercetin/catechin standard, sodium carbonate, gallic acid standards, ethanol, acetone, sterile polystyrene petri dish, sodium chloride, yeast extract, bacteriological peptone, and agar were all acquired from Sigma, Canada.

### 2.2 Cranberry juice preparation

A food processor was used to prepare the cleaned and defrosted cranberries, extracting the juice and then vacuum-filtering the cranberry juice prior to treatments. Before being used, the filtered cranberry juice was stored frozen at -18 degrees in sterile bottles.

### 2.3 Thermo-sonication unit set-up

An ultrasonic processor and a hot water bath were used to thermo-sonicate the cranberry juice samples of 100 mL each. The thermo-sonication of the cranberry juice was performed in a sterilized beaker using a probe ultrasonic processor (SONICS Vibra cell™, VCX 500, 500 W, 20 kHz, Newtown, Connecticut, USA). The defrosted cranberry juice was first brought to room temperature, measured into a beaker, then placed in a hot water bath with varying temperatures and durations as the cranberry juice samples underwent thermo and ultrasonication concurrently, using the following pulsed ultrasound parameters: energy of 120,000 J, amplitude of 60%, pulse ratio of 5 s (ON): 5 s (OFF), and temperatures of 35, 45, and 55 °C, and treatment durations of 1, 3, 5, and 7 minutes. The 13 mm probe was immersed to a fixed depth of approximately 25 mm in the 100 mL sample held in a jacketed beaker. Based on the nominal set energy of 120,000 J delivered to 100 mL, the corresponding volumetric energy density was approximately 1200 J/mL; because the instrument delivers a set total energy by automatically modulating the sonication time at the fixed 60% amplitude, the delivered energy and the effective treatment time were recorded for each run, and the sample temperature was monitored with a thermocouple and held at the target value (± 2 °C) by the surrounding ice bath. Throughout treatment, the ultrasonic probe was air-cooled to prevent overheating of the transducer, while the surrounding water bath maintained the target sample temperature.

#### 2.3.1 Color change measurement

The L*, a*, and b* values obtained from the chromameter (Model CR300, Konica-Minolta, USA) are used to measure the color changes of the thermo-sonicated treated cranberry juice samples. The following formula is used to estimate the total color change in terms of the Delta E value:

(1) $$\Delta E=\;\sqrt{{\left(L_{0}-L^{*}\right)}^{2}+({\left(a_{0}-a^{*}\right)}^{2}+{\left(b_{0}-b^{*}\right)}^{2}\;}\;\;\;\;\;\;$$

#### 2.3.2 Mineral content analysis

As described by[37], the concentrations of potassium (K), calcium (Ca), magnesium (Mg), phosphorus (P), sulfur (S), manganese (Mn), and iron (Fe) and other microminerals were determined from the thermo-sonicated treated samples which were first digested, and followed by inductively coupled plasma optical emission spectrometry (ICP-OES) analysis following the protocol proposed by [37].

#### 2.3.3 Ascorbic acid determination

Contents of ascorbic acids in the thermo-sonicated cranberry juice samples were determined using the method recommended by[38]. In brief, samples of cranberry juice are mixed with distilled water, ferric chloride (FeCl3), and 1,10-phenanthroline solutions, and agitated until a dark color change is produced. Using a spectrophotometer, the absorbance was measured at 430 nm[38].

#### 2.3.4 Total carotenoids analysis

To analyze total carotenoids, 5 ml of cranberry juice is combined with 0.8 ml of 80% (v/v) ethanol in a 10 ml tube and heated to 92°C for 30 min. The soluble heated extract is poured into 2-mL micro test tubes. To the remaining cranberry juice extract, 0.5 ml of 80% (v/v) ethanol is added and allowed to stand for 15 min at room temperature before vortexing. Following that, 0.4 ml of extract is mixed with 5 ml of 80% (v/v) acetone, and absorbance is measured at 663, 646, and 470 nm with a Synergy H4 hybrid microplate reader. Absorbances at 663 nm and 646 nm were used to correct for chlorophyll a (Ca) and chlorophyll b (Cb) co-extracted in the acetone/ethanol extract, so that the carotenoid estimate at 470 nm is not inflated by residual chlorophyll absorbance. Total carotenoids (Cx+c) were calculated in accordance with[39], [40]using the equation, where A470 is the absorbance at 470 nm and Ca and Cb are the chlorophyll a and b concentrations (µg/mL) obtained from the 663 nm and 646 nm readings:

(2) $$\text{Cx + c}\;=\left(1000{\text{A}}_{470}-3.27\text{Ca}\;-104\text{Cb}\right)/229.$$

#### 2.3.5 Antioxidant activity measurement

The ferric reducing antioxidant power (FRAP) assay and the 2,2-diphenyl-1-picrylhydrazyl (DPPH) radical scavenging test were used to determine the antioxidant activity of the treated samples. The DPPH radical scavenging activity of the samples was determined using the protocol of[41]. For this experiment, the sample extract was mixed with distilled water for the blank and freshly prepared 0.1 mM DPPH. The mixture was incubated in the dark for 20 min before measuring absorbance at 517 nm. The free radicals neutralized by DPPH radicals are given as an inhibition percentage and calculated using the equation:

(3) Inhibition(%)=(AbsControl@517nm-AbsSample@517nm)AbsControl@517nm

The FRAP measurement for the samples is performed using the approach suggested by[42]. To summarize, the FRAP reagent is freshly made by combining acetate buffer (pH 3.6), ferric chloride solution, and 2,4,6-tripyridyl-s-triazine (TPTZ) solution. The FRAP solution is then kept in a water bath at 37°C. Trolox acts as the standard, and distilled water as the blank. For the experiment, the prepared FRAP reagents are mixed with standard Trolox solution, sample extract, or a blank. The mixture is then incubated in the dark for 30 min then absorbance is measured at 593 nm. The antioxidant capacity of the samples is expressed as the FRAP value in millimoles of ferrous (Fe^2+^) equivalents per litre of sample (mmol Fe^2+^/L), determined against a ferrous sulphate calibration curve.

#### 2.3.6 Total phenolic content measurement

The total phenolic content of the treated cranberry samples is determined using the Folin-Ciocalteu method proposed by[43]. Briefly, the cranberry juice sample is combined with distilled water to make the blank and with Folin-Ciocalteu reagent for assessing the effect of processing on the cranberry juice. The mixture is allowed to rest for 5 min before 7.5% sodium carbonate solution is added to it and incubated for 90 min in the dark. Following incubation, absorbance is measured at 765 nm, and the total phenolic content of the sample is calculated using the gallic acid standard curve, expressed as mg gallic acid equivalent (GAE) per gram of fresh weight sample [43].

#### 2.3.7 Total anthocyanins content

The total anthocyanin content is determined using a pH differential method, as proposed by[44], [45]. The pH 1.0 buffer reagent was prepared with 980 mL of distilled water together with 1.86 g of potassium chloride (KCl). The pH was adjusted to 1.0 ± 0.05 with hydrochloric acid (HCl). Similarly, the pH 4.5, was prepared from 54.43 g of sodium acetate trihydrate (CH_3_CO_2_Na·3H_2_O) which was combined with distilled water in a beaker to the volume of 960 mL. After correcting the pH to 4.5 ± 0.05 with HCl, the buffer is diluted with 1 L of distilled water in a separate 1-L volumetric flask. The necessary protocol is followed to prepare the samples from the thermo-sonicated cranberry juice with each dilution's absorbance measured at 510 and 700 nm using a spectrophotometer [44], [45].

The total anthocyanin contents are determined using the equation:

(4) Anthocyaninpigmentcontent(mg/L)=(A∗MW∗DF∗1000)/ε

where, A = (Abs_510_ − Abs_700_) _pH 1_ − (Abs_510_ − Abs_700_) _pH 4.5,_ MW (cyanidin-3-glucoside) = 449.2 g/mol, DF = dilution factor, and molar absorptivity ε = 26 900 L mol^−1^ cm^−1^. Results were expressed as mg cyanidin-3-glucoside equivalent per litre (mg CGE/L).

#### 2.3.8 Total solids measurement

A digital refractometer (ATAGO, model PR32a, Tokyo, Japan) was used for measuring the total solids (brix) of the thermo-sonicated cranberry juice samples.

#### 2.3.9 LC-MS quantification of impact of non-thermal treatment on bioactive compounds

The thermo-sonicated treated cranberry juice samples were filtered using a 0.20 µm polytetrafluoroethylene (PTFE) syringe filter (Fisher Scientific) after extraction with methanol for liquid chromatography–mass spectrometry (LC-MS) analysis. The analyses were conducted using liquid chromatography coupled to electrospray-ionization quadrupole time-of-flight tandem mass spectrometry (LC–ESI–Q–TOF–MS/MS) to identify and monitor the two characteristic cranberry proanthocyanidin markers, procyanidin A1 and procyanidin B2, and to quantify treatment-related changes in their concentrations across the thermo-sonication conditions[46]. Compounds were identified from their accurate-mass precursor and product ions in negative-ionization mode and by comparison with authentic procyanidin A1 and B2 standards and were quantified against external calibration curves prepared from these standards. Separation used a reversed-phase C18 column and detection was performed in positive-ion mode; procyanidin A1 (C30H24O12) was detected at m/z 577.1341 (retention time ≈ 3.90 min) and procyanidin B2 (C30H26O12) at m/z 579.1497 (≈ 3.60 min). External calibration curves were linear over 10–2000 ng/mL for A1 and 10–500 ng/mL for B2, with limits of detection (LOD) of 4.88 and 2.42 ng/mL and limits of quantification (LOQ) of 16.3 and 8.1 ng/mL, respectively. These analytical parameters are summarised in Table S1, and representative extracted-ion chromatograms and mass spectra of both compounds in the standards and in a treated sample are shown in Fig. S1 (Supplementary Material).

#### 2.3.10 Analysis of microbial loads

Total microbial count was enumerated using the total plate count methodology. The thermo-sonicated cranberry juice samples were serially diluted in 0.9% saline water. The diluted juice samples were then spread on nutrient agar plates under sterile condition and incubated for 24 hours to assess the effect of the thermo-sonication treatment in reducing microbial loads in the cranberry juice.

### 2.4 Kinetic modeling

First order kinetics models were applied in the quantification of the rate of change of the studied physicochemical properties and were fitted to each parameter as a function of treatment duration. The rate of degradation model of the first-order kinetics is expressed as: Because several parameters displayed non-monotonic (biphasic) behaviour, the first-order model was used here as an empirical descriptor of the overall rate of change rather than as a mechanistic model. The coefficient of determination (R^2^) is reported for every fit; fits with low R^2^ are explicitly identified as poor and are interpreted with caution rather than over-interpreted, and parameters showing clear biphasic profiles (an initial change followed by a reversal) are flagged as not adequately captured by a single-exponential model. Full regression equations with 95% confidence intervals, residual diagnostics (Shapiro–Wilk normality and Durbin–Watson statistics), and formal model comparison by the Akaike Information Criterion (AIC) against biphasic (bi-exponential) and Weibull models are provided in the Supplementary Material (Tables S2–S4; Figs. S2–S3).

(5) $$C\left(t\right)\;=\;C_{0}\;\times \;exp\;\left(-k\;\times \;t\right)$$

Where C (t) is the concentration at time t (min), C_0_ is the initial concentration at t = 0 min, and k is the first-order kinetic rate constant (min^−1^). Linearization yields in (C/C_0_) = −k × t, and rate constants were estimated with linear regression. Half-lives (t½ = ln 2/k) and D-values (decimal reduction times; D = ln 10/k) were calculated for parameters exhibiting degradation kinetics (k > 0). Here “concentration” (C) denotes the measured value of the parameter of interest (e.g., TPC in mg GAE/100 mL, FRAP in mmol Fe^2+^/L, or TAC in mg CGE/L) at a given treatment time, normalised to its initial value C_0_. A negative k indicates net enhancement or accumulation of the parameter during treatment rather than degradation. The ultrasonication treatment at room temperature was modeled as thermo-sonication at 25°C.

The temperature sensitivity of the first-order degradation rate constants was quantified using the Arrhenius equation:

(6) k=A×exp(-Ea/RT)

where A is the pre-exponential (frequency) factor (min^−1^), Ea is the activation energy (kJ/mol), R is the universal gas constant (8.314 J·mol^−1^·K^−1^), and T is the absolute temperature, T, in kelvin (K). The linearized form, ln(k) = ln(A) − Ea/(RT), was used to estimate Ea and A by linear regression of ln(k) versus 1/T. The Q_10_ value (the factor by which k increases for a 10°C rise in temperature) and the z-value (the temperature change required for a 10-fold change in k) were also calculated as supplementary thermal sensitivity indices. Only treatment conditions exhibiting first-order degradation (k > 0) were included in the Arrhenius analysis.

### 2.5 Statistical analysis

All the analyses for the thermo-sonicated processed juice samples were conducted in triplicates, and the data were analyzed using one-way analysis of variance (ANOVA). The significant differences among the means treatments were determined using Tukey HSD at a significance level of p < 0.05. OriginPro 2024 (OriginLab Corporation, Northampton, MA, USA) was used for the statistical analysis.

## 3 Results and discussion

### 3.1 Overview and kinetics of chemical properties of thermosonicated cranberry juice

#### 3.1.1 Overview of the total flavonoid content (TFC) during thermo-sonication

The TFC of the control (untreated) sample is 6.85 ± 0.19 mg QE/100 mL (US-25 at 0 min). Following 1 min of treatment, TFC declined in US-25 and TS-35 ^o^C treatments (4.40 mg QE/100 mL) but remained elevated in TS-45°C (8.36 ± 2.30 mg QE/100 mL), which was significantly higher than other treatments (p < 0.05) (Table 1). Tukey HSD analysis at t = 1 min revealed that TS-45 ^o^C was significantly different from US-25 and TS-35 ^o^C (F = 5.503, HSD = 3.71).

**Table 1:** Total Flavonoid Content (TFC) of thermo-sonicated cranberry juice at different treatment time.

**Treatment**	**t = 0 min**	**t = 1 min**	**t = 3 min**	**t = 5 min**	**t = 7 min**
US-25	6.85 ± 0.19 a	4.40 ± 0.22 b	9.74 ± 0.62 a	7.07 ± 2.32 a	6.24 ± 0.87 a
TS-35	6.85 ± 0.19 a	4.40 ± 0.22 b	7.32 ± 0.75 a	4.24 ± 0.39 b	7.56 ± 0.12 a
TS-45	6.85 ± 0.19 a	8.36 ± 2.30 a	8.36 ± 2.30 a	4.94 ± 0.41 b	9.29 ± 3.82 a
TS-55	6.85 ± 0.19 a	6.62 ± 1.64 ab	6.62 ± 1.64 b	4.50 ± 0.16 b	5.01 ± 0.38 a

*t = 0 represented the untreated sample, and US-25 represented the ultrasound-only (sonication) control at room temperature (measured as 25 °C). Different lowercase letters within a column indicate significant differences (Tukey HSD, p < 0.05)*.

The total flavonoid content (TFC) in the ultrasound only (US-25 ^o^C) treatment samples increased at 3 min (9.74 ± 0.62 mg QE/100 mL), attributable to released flavonoids bound made available through cavitation induced breakdown of flavonoid-polysaccharide complexes in the cranberry juice matrix. The known phenomenon, which is reported in other berry matrices subjected to ultrasonication, is referred to as sonochemical extraction. However, the combination of mild heat (thermo-sonication) treatments for 3 and 5 min resulted in lower total flavonoids contents (TFC) as shown on Table 1, which may be potentially due to flavone aglycone degradation from the thermal treatment. The values of the 7 min treatment were not statistically different from the other 35 ^o^C thermo-sonicated samples (p > 0.05) indicating flavonoid pools convergence at extended (7 min) treatment duration. These trends are consistent with reports for other sonicated berry juices, in which ultrasonic cavitation first liberates matrix-bound flavonoids (sonochemical extraction) before prolonged or heated treatment promotes their degradation; for example, thermo-sonication increased the flavonoid content of blueberry juice by up to approximately 250% relative to raw juice[47], and comparable cavitation-driven release has been reported for blackcurrant and blackberry juices[22], [31].

#### 3.1.2 Kinetics of total flavonoids contents

Total flavonoid content did not conform to first-order degradation kinetics in most treatments, with negative apparent rate constants (indicating net enhancement) observed for US-25, TS-35, and TS-45 (Fig. 1). However, TS-55°C, exhibited a first-order degradation (k = 0.057 min^−1^; R^2^ = 0.714; t½ = 12.19 min). The oscillatory behavior of total phenolic content (TFC), that is initially decreasing followed by a recovery, then a second decrease, implies competing matrix bonds release and degradation mechanisms operating at different duration scales, which is consistent with biphasic kinetics.

**Fig. 1:**
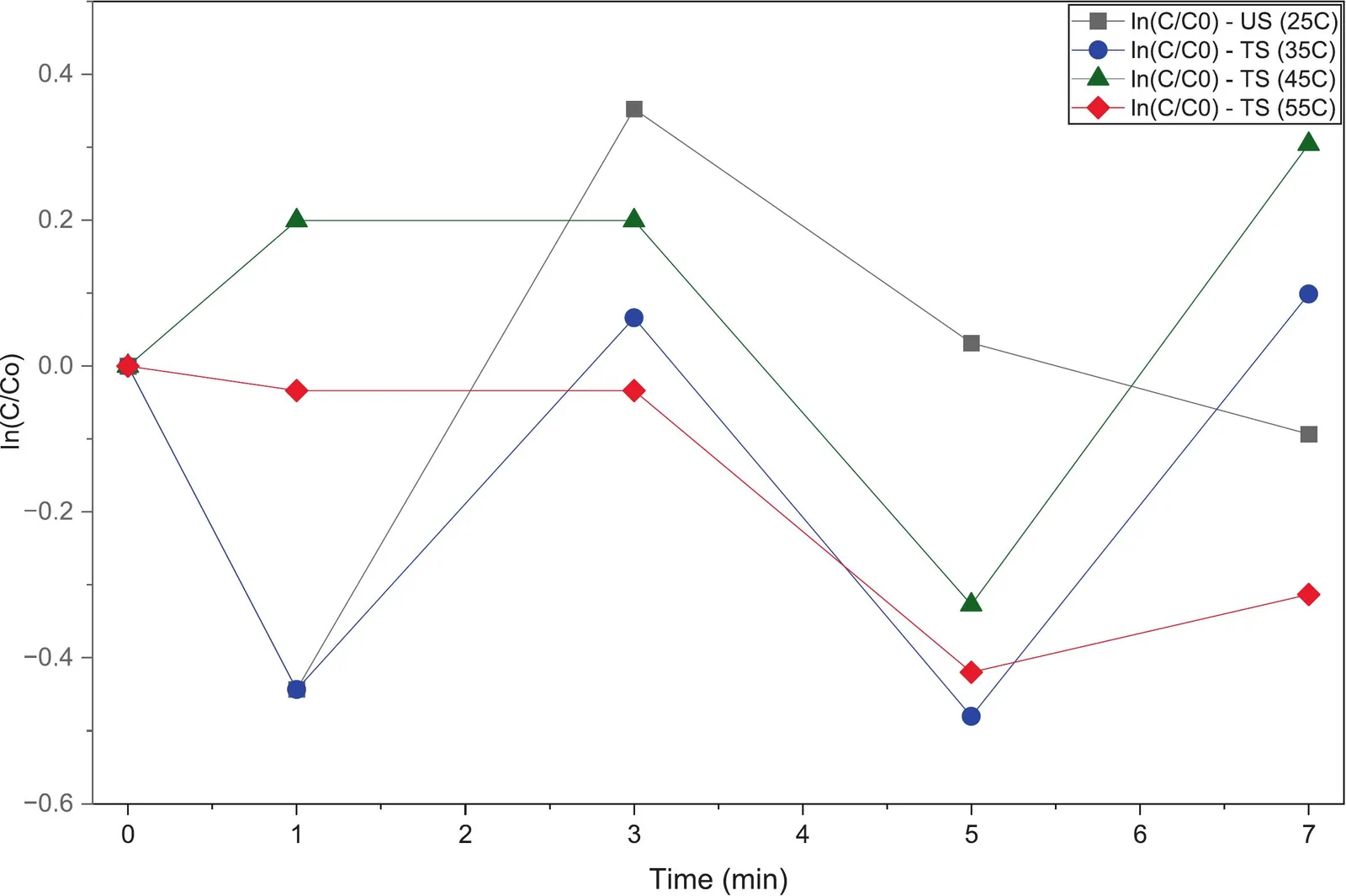
First-order kinetics of Total Flavonoids Content degradation in cranberry juice during thermo-sonication.

#### 3.1.3 Overview of total phenolic content (TPC) during thermo-sonication

The total phenolic content (TPC) at different treatment time for ultrasonicated and thermo-sonicated samples is shown in Table 2. The 35°C thermo-sonicated control sample was 7.99 ± 0.23 mg GAE/100 mL, while the ultrasonication (TS-25) control sample was 9.59 ± 0.27 mg GAE/100 mL, which were showed a compositional difference (p < 0.05). The ultrasound (TS-25) treated samples proved a distinguishing biphasic kinetic profile, that is, an initial decrease to 4.01 ± 0.11 mg GAE/100 mL at 1 min, followed by a surge to a peak of 12.85 ± 0.38 mg GAE/100 mL at 3 min (US-3), was observed, which then stabilized at 11 mg GAE/100 mL with 7 min (US-7) of treatment. The thermo-sonicated treatments samples, that is TS-35, TS-45, and TS-55 presented a more constant but decreased total phenolics content (TPC) profile, decreasing speedily after 1 min and being significantly lower than the ultrasound (TS-25) treated samples at all subsequent treatment durations (F values ranging from 100.1 to 383.4; all p < 0.001). The thermo-sonicated sample had 3.07–3.69 mg GAE/100 mL for 7 min treatment, while, the ultrasonicated sample had a retention of 10.97 ± 0.14 mg GAE/100 mL, hence a 67-72% difference. The excessive phenolic content degradation was because of polyphenol thermo-acoustic stress, where thermo-sonication treatment effects through sonochemical radical creation and heat-induced oxidation, which is consistent with first order kinetics degradation causes the decline. Thermo-sonication 55°Cfor 3 min treatment (TS-55:3min) presented the lowest total phenolic content (TPC) with 5.13 ± 0.13 mg GAE/100 mL; significantly lower than TS-35:3 and TS-45:3, p < 0.05, which confirms the temperature-dependent phenolic compound degradation. The apparent difference between the ultrasound control (9.59 ± 0.27 mg GAE/100 mL) and the thermo-sonication control (7.99 ± 0.23 mg GAE/100 mL) at t = 0 arises because the ultrasound and thermo-sonication experiments were performed on separate juice batches prepared from different lots of frozen cranberries. Within each experiment, every treatment was compared against its own concurrently measured untreated control, so comparisons are made within, and not across, batches; when the data are expressed as relative retention (C/C_0_) the two data sets follow the same qualitative pattern. This biphasic behaviour of the ultrasound-only samples, an initial decline followed by a cavitation-driven rise in extractable phenolics, mirrors observations in sonicated apple, tomato and blueberry juices, in which moderate ultrasonication enhances phenolic recovery whereas the additional thermal load of thermo-sonication shifts the balance towards oxidative degradation (Li et al., 2025; [22], [47].

**Table 2:** Total Phenolic Content (TPC) of thermo-sonicated cranberry juice measured in mg GAE/100 mL.

**Treatment**	**t = 0 min**	**t = 1 min**	**t = 3 min**	**t = 5 min**	**t = 7 min**
US-25	9.59 ± 0.27 a	4.01 ± 0.11 a	12.85 ± 0.38 a	11.16 ± 0.19 a	10.97 ± 0.14 a
TS-35	7.99 ± 0.23 b	3.18 ± 0.09 b	6.44 ± 0.16 b	3.13 ± 0.25 b	3.07 ± 0.01 b
TS-45	7.99 ± 0.23 b	3.11 ± 0.04 b	6.73 ± 1.05 b	3.28 ± 0.13 b	3.08 ± 0.11 b
TS-55	7.99 ± 0.23 b	3.07 ± 0.06 b	5.13 ± 0.13 c	3.65 ± 0.60 b	3.69 ± 0.70 b

*t=0 represented untreated samples and US-25 represented ultrasonication at room temperature (measured to be 25°C). Different lowercase letters within a column indicate significant differences (Tukey HSD, p < 0.05)*.

#### 3.1.4 Kinetics of phenolics degradation

Treatments at 25°C produced a negative rate (k = −0.081 min^−1^) for the degradation kinetics, which reflects a net improvement of the extractable phenolics via acoustic cavitation mediated disruption of polyphenol-matrices interactions. Conversely, all thermo-sonicated treated samples yielded positive rate constants, implying net phenolics degradation: TS-35°C (k = 0.099 min^−1^; t½ = 6.99 min), TS-45°C (k= 0.095 min^−1^; t½ = 7.30 min), and TS-55°C (k = 0.066 min^−1^; t½ = 10.52 min). Particularly, the moderate R^2^ values (0.25–0.38) for total phenolic content (TPC) indicate that the degradation kinetics of phenolic during thermo-sonication are not exclusively first-order, possibly, indicating competing extraction and degradation mechanisms (Fig. 2). A decline in the rate of degradation of the total phenolic content (TPC) was observed with increasing temperatures (k decreased from 0.099 at 35°C to 0.066 at 55°C). The D-value correspondingly increased from 23.23 min (35°C) to 34.96 min (55°C), indicating greater phenolic preservation at higher thermo-sonication temperatures despite the expected thermal sensitivity of polyphenols.

**Fig. 2:**
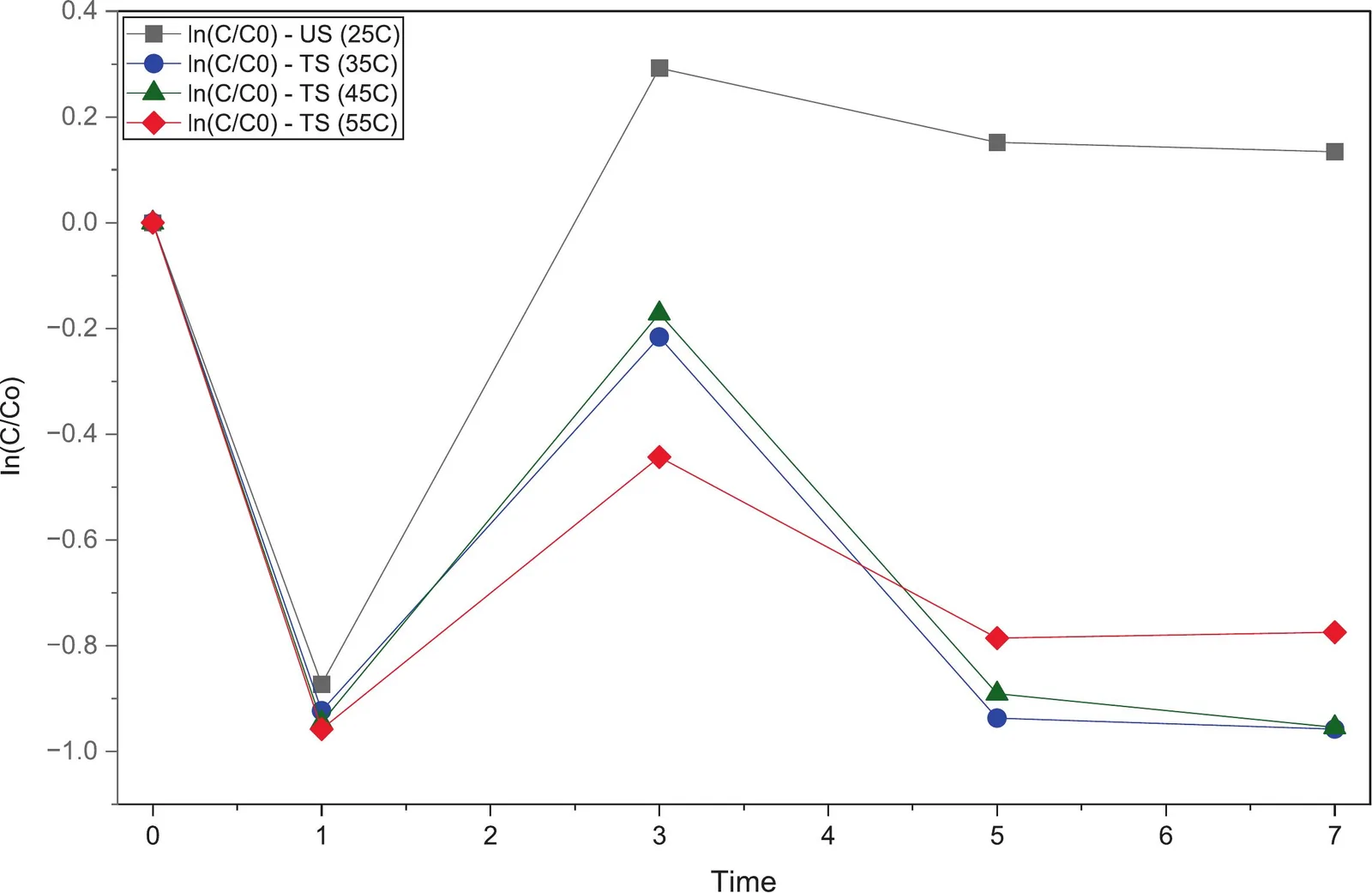
First-order kinetics of total phenolic content degradation in cranberry juice during thermo-sonication.

#### 3.1.5 Overview of FRAP antioxidant capacity during thermo-sonication

The FRAP assay analysis of the thermo-sonicated samples showed a noticeable and treatment-specific kinetic pattern (Table 3). For the US treatment, FRAP declined monotonically from 0.57 ± 0.07 mmol Fe^2+^/L at t = 0 to 0.24 ± 0.01 mmol Fe^2+^/L at 7 min, reflecting the net oxidative burden of prolonged ultrasonic treatment. In sharp contrast, thermo-sonication at 35°C (TS-35) exhibited a consistent and significant increase in FRAP capacity throughout the treatment duration: from 0.64 ± 0.10 mmol Fe^2+^/L at t = 0 to 0.93, 1.21, 1.50, and 1.78 ± 0.10 mmol Fe^2+^/L at 1, 3, 5, and 7 min, respectively representing a 2.77-fold enhancement over the treatment course. Tukey HSD analysis confirmed that TS-35 was significantly different from all other treatments at each time point from t = 1 min onwards (p < 0.001; F values: 105.0 to 698.6).

**Table 3:** FRAP Antioxidant Capacity (mmol Fe^2+^/L) of thermo-sonicated cranberry juice.

**Treatment**	**t = 0 min***(mmol Fe^2+^/L)*	**t = 1 min***(mmol Fe^2+^/L)*	**t = 3 min***(mmol Fe^2+^/L)*	**t = 5 min***(mmol Fe^2+^/L)*	**t = 7 min***(mmol Fe^2+^/L)*
US-25	0.57 ± 0.07 a	0.34 ± 0.02 b	0.30 ± 0.01 c	0.26 ± 0.04 d	0.24 ± 0.01 d
TS-35	0.64 ± 0.10 a	0.93 ± 0.10 a	1.21 ± 0.10 a	1.50 ± 0.10 a	1.78 ± 0.10 a
TS-45	0.57 ± 0.07 a	0.38 ± 0.04 b	0.38 ± 0.04 b	0.41 ± 0.01 b	0.30 ± 0.02 b
TS-55	0.57 ± 0.07 a	0.25 ± 0.01 b	0.25 ± 0.01 c	0.29 ± 0.04 cd	0.31 ± 0.01 b

*t=0 represented untreated samples and US-25 represented ultrasonication at room temperature (measured to be 25°C). Different lowercase letters within a column indicate significant differences (Tukey HSD, p < 0.05)*.

The observed increase in the antioxidant activity with the TS-35°C treatment was possibly due to the release of phenolic bonds from the cranberry juices matrices, due to the thermodynamic energy breaking of hydrogen bonds among cell wall polysaccharides and phenolics without resulting in compounds degradation. Products of Maillard reaction created under mild heat treatments can also contribute to the reducing capacity. The combined thermo-acoustic treatment appears to function synergistically at 35°C, reducing compounds faster than they are degraded. At higher temperatures (TS-45 and TS-55), FRAP values were comparably lower and similar to ultrasound (US) treatment, suggesting that at 45–55°C, thermal degradation outpaces the release mechanism. A treatment-induced increase in ferric-reducing capacity of this kind has also been reported for thermo-sonicated blackberry and blueberry juices, in which mild sonication liberated additional reducing phenolics and Maillard-type products and raised the antioxidant capacity above that of the untreated juice[31], [47].

#### 3.1.6 Kinetics of antioxidant activity

FRAP antioxidant capacity exhibited divergent kinetics depending on temperature (Table 3 and Fig. 3). The ultrasound-only treatment at 25°C showed degradation (k = 0.101 min^−1^; R^2^ = 0.748), while thermo-sonication at 35°C uniquely produced a strong enhancement (k = −0.136 min^−1^; R^2^ = 0.937), consistent with the progressive release of reducing compounds as discussed in section 3.1.3. At 45°C and 55°C, FRAP showed degradation but at decreasing rates (k = 0.063 and 0.047 min^−1^, respectively).

**Fig. 3:**
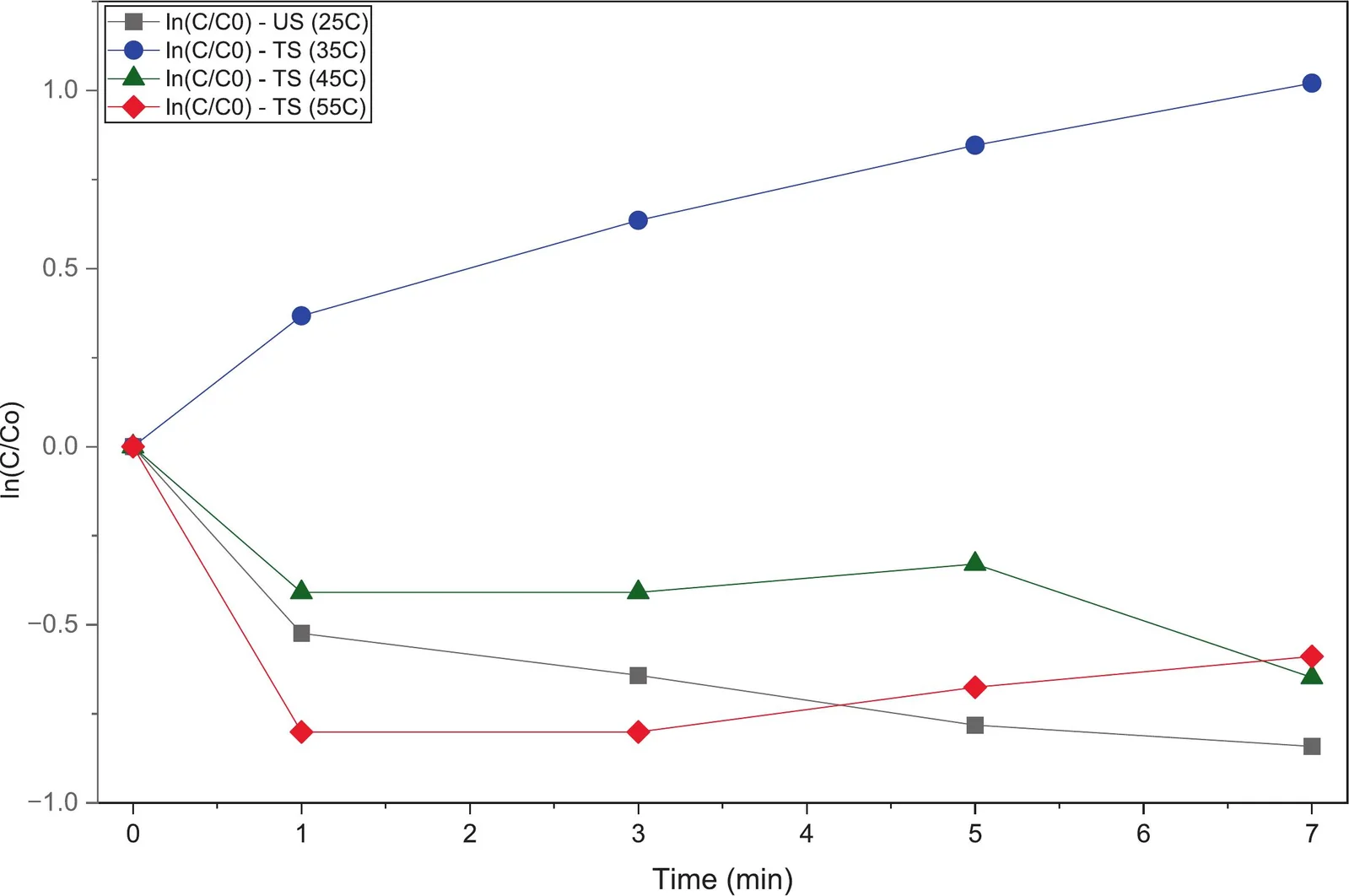
First-order kinetics of FRAP Antioxidant Capacity degradation in cranberry juice during thermosonication.

#### 3.1.7 Overview of DPPH radical scavenging activity during thermo-sonication

DPPH radical scavenging activity showed complex, treatment-dependent kinetics with all pairwise differences being statistically significant (p < 0.001) at each time point due to high measurement precision within replicates (Table 4).

**Table 4:** DPPH Radical Scavenging Activity (%) of thermo-sonicated cranberry juice.

**Treatment**	**t = 0 min**	**t = 1 min**	**t = 3 min**	**t = 5 min**	**t = 7 min**
US	32.50 ± 7.26a	12.94 ± 2.81a	16.74 ± 1.33b	16.48 ± 2.71b	23.97 ± 4.02a
TS-35	32.50 ± 7.26a	16.74 ± 1.33a	19.28 ± 0.46b	23.43 ± 2.26a	13.51 ± 1.80a
TS-45	32.50 ± 7.26a	10.82 ± 2.23a	26.25 ± 3.08a	1.02 ± 1.61c	13.79 ± 8.04a
TS-55	32.50 ± 7.26a	7.50 ± 2.91b	23.57 ± 2.09a	11.63 ± 3.38b	12.17 ± 1.18a

*t=0 represented untreated samples and US-25 represented ultrasonication at room temperature (measured to be 25°C). Different lowercase letters within a column indicate significant differences (Tukey HSD, p < 0.05)*.

The untreated control showed a DPPH radical-scavenging activity of 32.50 ± 7.27% inhibition (Table 4). At 1 min, ultrasound (US-1) decreased scavenging activity to 12.94 ± 2.81%, with a partial recovery to 23.97 ± 4.02% at 7 min. Thermo-sonication at 55 °C (TS-55, 1 min) gave the lowest early value (7.50 ± 2.91%), reflecting temperature-dependent degradation of phenolic and anthocyanin scavengers, while the anomalously low value at TS-45, 5 min (1.02 ± 1.61%) is consistent with a transient loss of antioxidant capacity, possibly linked to time–temperature interaction and structural alteration of antioxidant compounds. Results are reported as % inhibition to remain consistent with the method described in Eq. (3); the corresponding ascorbic-acid-equivalent values (mg AAE/L) followed the same trends. The DPPH values declined across all the treated samples suggesting that the thermo-sonication treatments, at higher temperatures negatively affected DPPH activity. The decline in radical-scavenging activity at higher temperatures is in line with reports that the DPPH activity of sonicated juices decreases once the thermal contribution dominates, whereas ambient ultrasonication is comparatively protective of antioxidant constituents[48], [22].

#### 3.1.8 Kinetics of DPPH radical scavenging activity

DPPH radical scavenging activity followed first-order degradation across all temperatures, but with substantial variability in the fit quality (Fig. 4). The highest rate constant was observed at 45°C (k = 0.213 min^−1^; t½ = 3.25 min), which is markedly faster than at other temperatures and reflects the anomalously low DPPH value recorded at the 5-min treatment point (5.18 mg AAE/L). The ultrasound (US) treatment at 25°C showed the slowest degradation (k = 0.007 min^−1^; D = 340.36 min), indicating that radical scavenging compounds in cranberry juice are more resistant to sonic degradation at ambient temperature than to a combined thermo-acoustic stress.Fig 5.Fig 6.

**Fig. 4:**
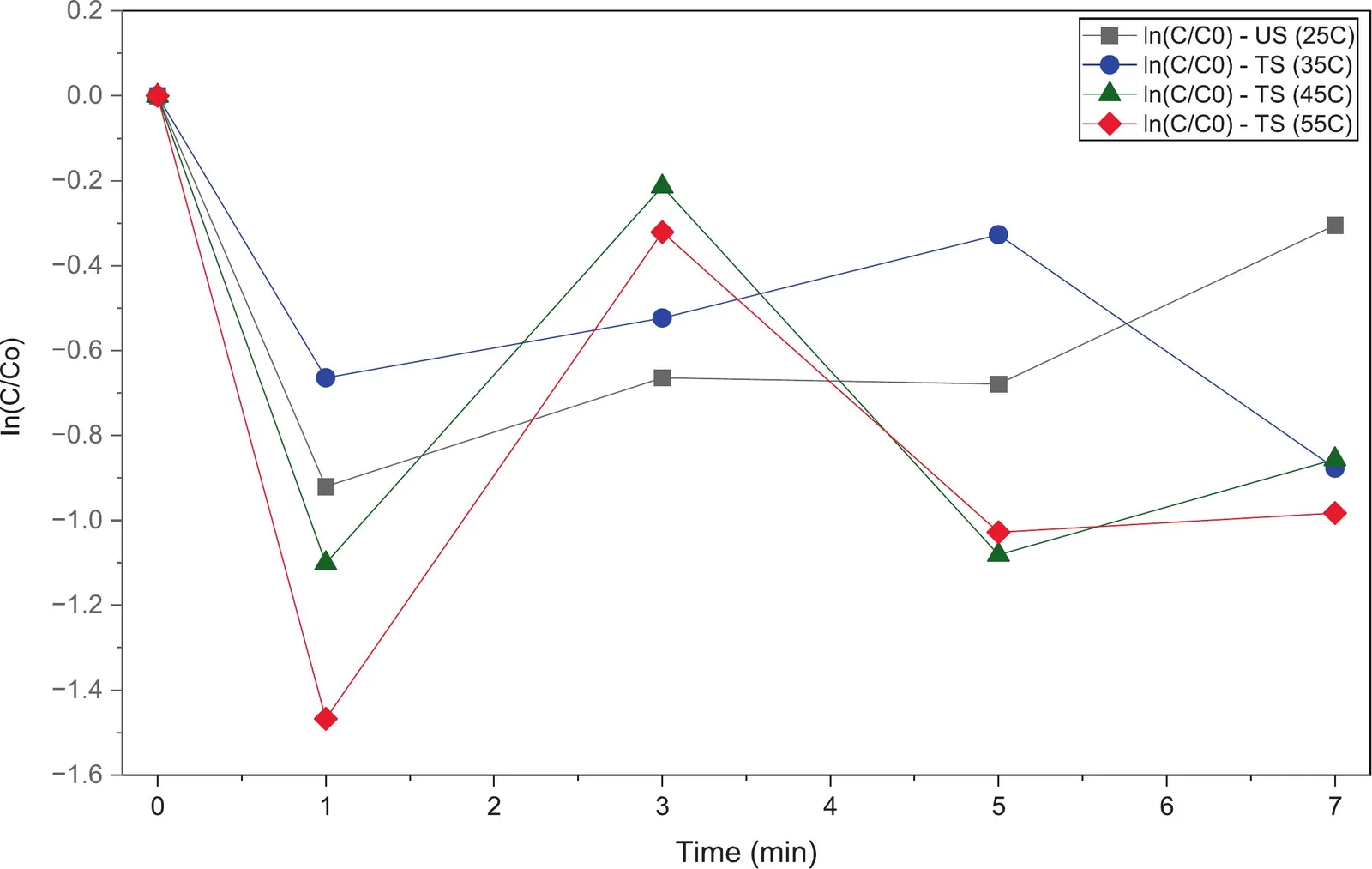
First-order kinetics of DPPH Radical Scavenging Activity degradation in cranberry juice during thermo-sonication.

**Fig. 5:**
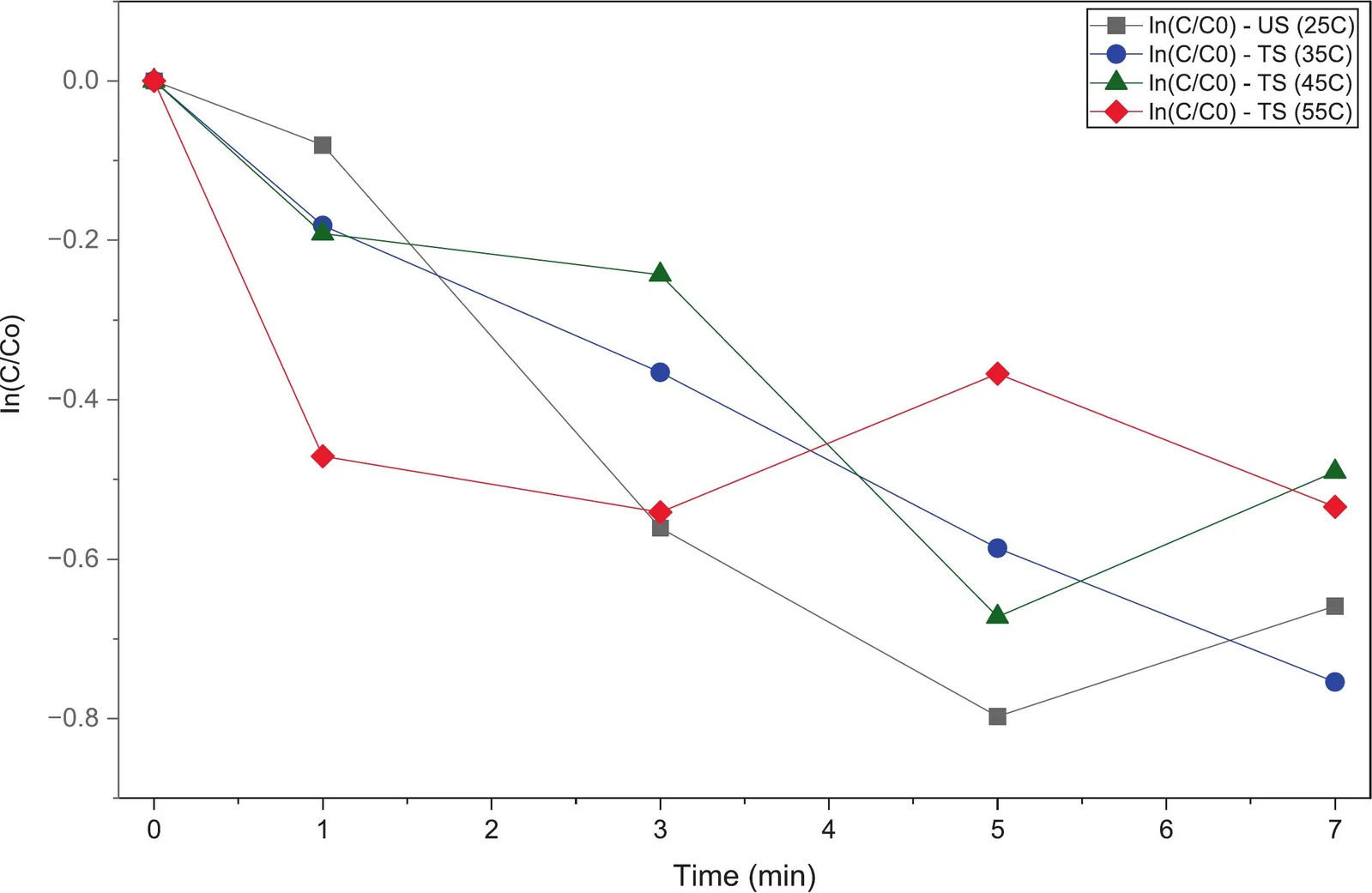
First-order kinetics of Total Anthocyanin Content degradation in cranberry juice during thermo-sonication.

**Fig. 6:**
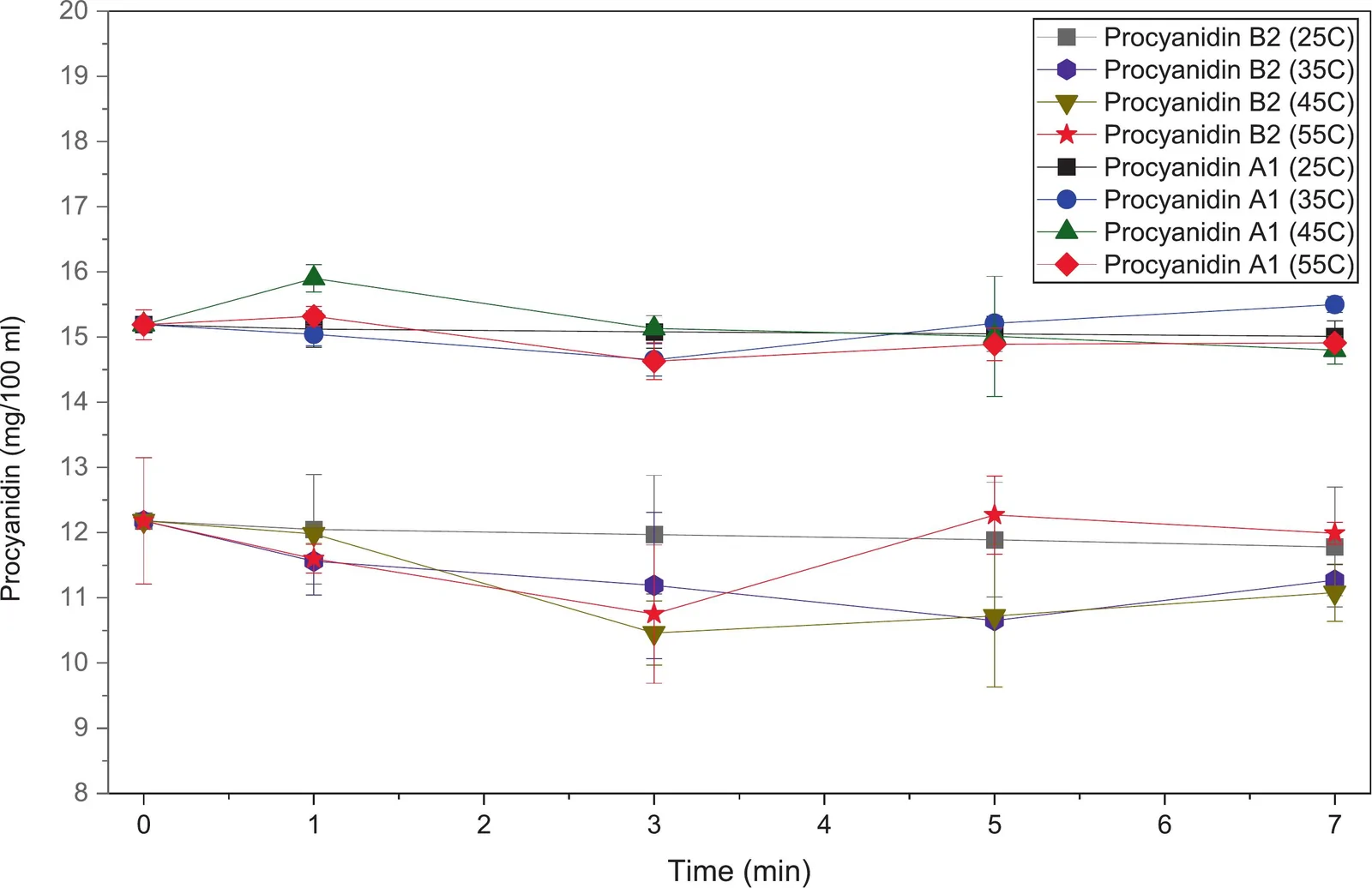
Procyanidin B2 and A1 variation during thermo-sonication.

#### 3.1.9 Overview of total anthocyanin content (TAC) during Thermo-sonication

The total monomeric anthocyanin content (TAC), determined by the pH-differential method, is reported in Table 5. The untreated control contained 35.38 mg CGE/L. TAC varied considerably across treatments and treatment times, and because of substantial replicate variability most differences among the US, TS-35, TS-45 and TS-55 treatments within a given treatment time were not statistically significant (Tukey HSD, p > 0.05; Table 5). For the ultrasound treatment, an apparent early rise at 1 min (US-1, 130.67 mg CGE/L) was followed by lower values at 5 and 7 min (US-5, 19.97; US-7, 21.31 mg CGE/L). All values in this section correspond to those reported in Table 5.

**Table 5:** Total Anthocyanin Content (mg CGE/L) of thermo-sonicated cranberry juice.

**Treatment**	**t = 0 min**	**t = 1 min**	**t = 3 min**	**t = 5 min**	**t = 7 min**
US	35.38 ± 0.19a	130.67 ± 5.68a	23.80 ± 1.31c	19.97 ± 2.55c	21.31 ± 0.33c
TS-35	35.38 ± 0.19a	138.18 ± 13.28a	28.97 ± 5.19c	105.45 ± 10.51b	130.70 ± 6.57a
TS-45	35.38 ± 0.19a	116.40 ± 18.68a	32.11 ± 5.00c	90.35 ± 3.12b	107.83 ± 5.23ab
TS-55	35.38 ± 0.19a	102.20 ± 47.27a	29.79 ± 7.01a	11.67 ± 1.10a	125.75 ± 25.58a

*t=0 represented untreated samples and US-25 represented ultrasonication at room temperature (measured to be 25°C). Different lowercase letters within a column indicate significant differences (Tukey HSD, p < 0.05)*.

At the longest treatment time (7 min), the thermo-sonicated samples retained higher TAC than the ultrasound-only sample (TS-35, 130.70; TS-55, 125.75; TS-45, 107.83 mg CGE/L; versus US-7, 21.31 mg CGE/L), and at this time point US-7 was significantly lower than the thermo-sonicated samples (Tukey HSD, p < 0.05; Table 5). At the earlier time points the between-treatment differences were not statistically significant, so the present data do not identify a single optimal temperature for anthocyanin retention; they indicate mainly that combining mild heat with sonication tended to preserve more anthocyanin at 7 min than sonication alone. Given the high replicate variability, this anthocyanin results should be interpreted with caution[8]. On the basis of these data alone, and given the high replicate variability, a single optimal temperature for anthocyanin retention cannot be firmly established; the thermo-sonicated samples nonetheless tended to retain more anthocyanin than ultrasound alone at the longest treatment time. The greater anthocyanin retention under combined mild heat and sonication, relative to sonication alone, is consistent with thermo-sonication studies on blueberry and pomegranate-type systems, in which controlled mild heating stabilises the coloured flavylium form and limits cavitation-driven pigment loss[8], [47].

#### 3.1.10 Kinetics of anthocyanin degradation

The total anthocyanin content (TAC) showed constant first-order degradation attributed to the temperature changes employed in the study, that is, 25°C, 35°C, 45°C and 55°C. Treatment at 35°C gave the best overall first-order match (R^2^ = 0.989), with a constant rate of k = 0.105 min^−1^ corresponding to a half-life of 6.59 min and a D-value of 21.90 min. Ultrasound treatments at 25 °C had high rates of degradation (k = 0.111 min^−1^; t½ = 6.23 min; R^2^ = 0.795), suggesting that, at even at low temperatures, acoustic cavitation mediated anthocyanin destruction can occur.

A striking inverse temperature dependence was observed: the degradation rate constant decreased progressively from k = 0.111 min^−1^ at 25°C to k = 0.047 min^−1^ at 55°C, with the D-value increasing from 20.69 to 48.79 min. At 55°C, the reduced cavitation intensity apparently outweighs the additional thermal degradation, yielding a net reduction in the overall rate constant.

### 3.2 Temperature sensitivity of chemical properties of thermosonicated cranberry juice

The temperature sensitivity (presented as Arrhenius parameters) of the chemical properties of thermosonicated cranberry juice is presented in Table 6.

**Table 6:** Arrhenius parameters for chemical properties.

**Parameter**	**Temperatures Used**	**Ea (kJ/mol)**	**A (min^−1^)**	**R^2^**	**Q_1_**_0_	**z-value (°C)**
**TPC**	35, 45, 55	−17.0	1.36×10^−4^	0.813	0.81	−106.7
**FRAP**	25, 45, 55	−20.6	2.53×10^−5^	0.992	0.78	−88.3
**DPPH**	25, 35, 45, 55	59.4	3.37×10^8^	0.615	2.17	−31.6
**TAC**	25, 35, 45, 55	−23.0	1.19×10^−5^	0.865	0.75	−79.2

The Arrhenius equation k = A × exp(−Ea/RT) was linearized as ln(k) = ln(A) − Ea/(RT) and fitted to the temperature-dependent rate constants. Q_10_ (rate factor per 10°C) and z-value (temperature for a 10-fold rate change) were calculated as supplementary indices. Only conditions with k > 0 were included.

#### 3.2.1 Temperature sensitivity of phenolic degradation

TPC degradation under thermo-sonication (35–55°C) yielded a negative apparent activation energy (E_a_ = −17.03 kJ/mol; R^2^ = 0.813; Q_10_ = 0.81) (Table 6), indicating that phenolic degradation is also primarily driven by cavitation-mediated processes. The z-value of −106.7°C reflects the relatively weak temperature sensitivity of the phenolic degradation rate over the 35–55°C range.

#### 3.2.2 Temperature sensitivity of FRAP antioxidant capacity degradation

FRAP degradation showed the strongest negative apparent activation energy (Ea = −20.59 kJ/mol; R^2^ = 0.992; Q_10_ = 0.78), as shown in Table 6, with the Arrhenius fit being remarkably linear across the three degradation-exhibiting temperatures (25, 45, 55°C; The Arrhenius model describes degradation (k > 0); the 35 °C FRAP data showed a negative rate constant (net enhancement) and therefore cannot be placed on a degradation Arrhenius plot.). The near-perfect linearity (R^2^ = 0.992) of the FRAP Arrhenius plot strongly supports the interpretation that a single rate-determining step governed by cavitation intensity rather than thermal energy controls the loss of reducing power during thermo-sonication.

#### 3.2.3 DPPH temperature sensitivity

The temperature dependence of DPPH radical-scavenging activity follows an Arrhenius pattern with a positive activation energy of Ea = 59.4 kJ/mol (R^2^ = 0.615; Q_10_ = 2.17; z-value = -31.6°C), as shown in Table 6, implying that degradation of antioxidant is enhanced significantly through thermal energy. The Q_10_ of 2.29 indicates the degradation rate doubles approximately for each 10°C increases, which is consistent with the thermal sensitivity of radical scavenging compounds such as anthocyanins and vitamin C in juices from berries. On the contrary, the moderately fit quality (R^2^ = 0.545) and the constant high rate of TS-45°C with k = 0.213 min^−1^ implies that DPPH kinetics are more complex than just a temperature-dependent first-order process. The z-value of 26.9°C of DPPH is in the expected range for thermal degradation (20 - 40°C) of antioxidant components in juices from fruits, different from the negative z-values noted for cavitation-dominated parameters.

#### 3.2.4 Anthocyanin temperature sensitivity

The Arrhenius plot for TAC degradation across all four temperatures (25–55°C) yielded an apparent activation energy of Ea = −22.97 kJ/mol (R^2^ = 0.865), with Q_10_ = 0.75 and z-value = −79.2°C (Table 6). The negative Ea is thermodynamically significant: it indicates that the overall anthocyanin degradation rate decreases with increasing temperature under thermo-sonication conditions. This is in direct contrast to pure thermal degradation of anthocyanins, which typically shows positive Ea values of 50–100 kJ/mol. The Q_10_ value of 0.75 shows that for every 10°C increases in thermo-sonication temperature, the total anthocyanin content (TAC) degradation rate decreases nearly by 25%. This finding is highly relevant for process design: it implies that thermo-sonication at moderately elevated temperatures (45–55°C) is paradoxically more protective of anthocyanins than processing at lower temperatures, because the diminished cavitation intensity at higher temperatures reduces the primary degradation mechanism.

### 3.3 Primary bioactive compounds: procyanidin A1 and B2

#### 3.3.1 Overview of procyanidin A1 and B2

The A-type and B-type procyanidins are the signature bioactive constituents of cranberry juice, with Procyanidin A1 (a doubly linked A-type dimer) and Procyanidin B2 (an epicatechin dimer with a single B-type interflavan bond) serving as markers of cranberry authenticity and bioactivity. Their concentrations under thermo-sonication conditions are presented in figure 6.

Procyanidin B2 concentrations ranged from 10.46 ± 0.49 to 12.27 ± 0.60 mg/100 mL across all thermo-sonication temperatures and time points, while Procyanidin A1 ranged from 14.63 ± 0.28 to 15.90 ± 0.21 mg/100 mL (individual treatment means are reported in the procyanidin data of section 3.6 and figure 6). Critically, ANOVA revealed no statistically significant differences in either procyanidin concentrations attributable to the thermo-sonication temperature (35°C, 45°C, or 55°C) or treatment duration (1-7 min) (p > 0.05 in all cases; Tukey compact letter display (CLD): all treatments assigned letter “a”). This is among the most significant findings of this study: thermo-sonication does not significantly degrade the hallmark A-type procyanidins of cranberry juice under any of the conditions investigated. The thermal stability of cranberry procyanidins has been partially attributed to their highly condensed, double linked A-type interflavanic bond structure, which confers greater resistance to hydrolytic and oxidative cleavage compared to B-type procyanidins found in other fruits. The higher concentration of procyanidin A1 relative to procyanidin B2 is consistent with the known chemistry of cranberry, in which A-type (doubly linked) procyanidins predominate over B-type dimers; this predominance is a distinguishing chemotaxonomic feature of cranberry relative to most other fruits and supports the compound assignment[49].

The ultrasonication generated acoustic cavitations during processing of the cranberry juice can cleave interflavanic bonds via radical processes but the results suggest that with the ultrasound frequencies and amplitudes of 20 kHz, 60% amplitude, employed, such degradation was not observed with the 7 min treatment window. The study findings hence support the feasibility of the processing technology of thermo-sonication to achieve the objectives of microbial inactivation without compromising the key bioactive components of cranberry juice.

#### 3.3.2 Kinetics of procyanidin A1 and B2

First-order kinetics modeling was applied to the two major A-type and B-type proanthocyanidins identified in cranberry juice: procyanidin B2 (a B-type dimer, epicatechin–(4β→8)–epicatechin) and procyanidin A1 (an A-type dimer, epicatechin (2β→O→7, 4β→8)–epicatechin). These dimeric proanthocyanidins are of particular interest because A-type procyanidins are characteristic biomarkers of cranberry and confer anti-adhesion properties against uropathogenic bacteria. Since no time-zero measurements were available for the procyanidins (measurements began at t = 1 min), the 1-minute values were used as the reference concentration (C_0_) for kinetics fitting. Thermo-sonication was applied at three temperatures (35, 45, and 55°C), with treatment times of 1, 3, 5, and 7 min. The kinetics results are presented in Fig. 7.

**Fig. 7:**
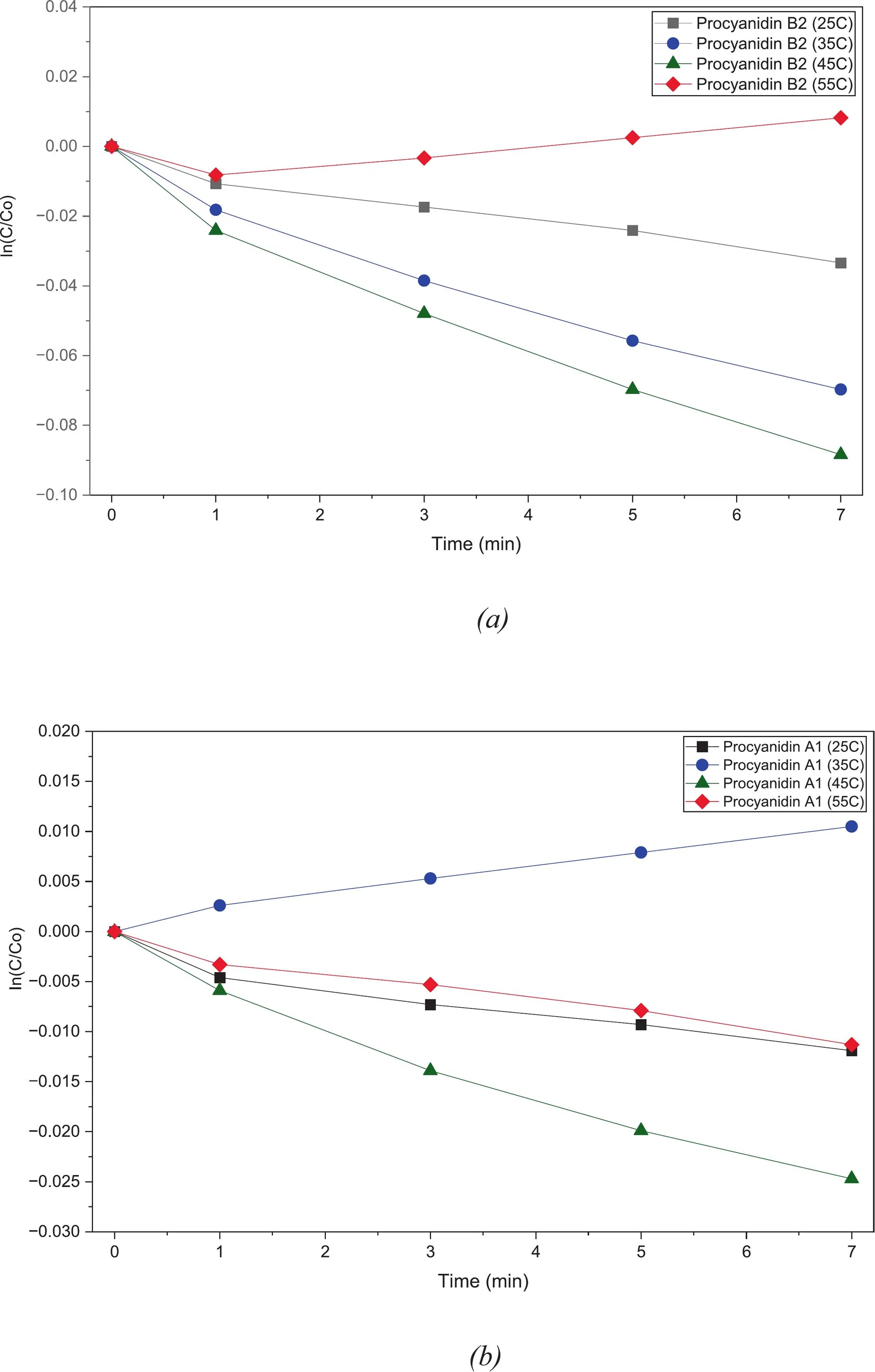
First-order kinetics of procyanidin (a) B2 and (b) A1 degradation in cranberry juice during thermo-sonication.

The grand mean of procyanidin B2 is 11.29 ± 0.57 mg/100 mL, with all treatment conditions showing near-zero rate coefficients for the three temperatures used: k = 0.006 min^−1^ at 35°C (R^2^ = 0.224), k = 0.010 min^−1^ at 45°C (R^2^ = 0.210), and k = −0.012 min^−1^ at 55°C (R^2^ = 0.268).

The total change across the full treatment duration was less than 8% for all conditions (−2.51% at 35°C, −7.51% at 45°C, +3.36% at 55°C), with an overall coefficient of variation (CV) of only 5.0%. The low R^2^ values reflect a poor fit to the first-order model; this is not due to complex kinetics but rather time trend in the data. The variation in B2 concentration across time points is comparable to analytical measurement uncertainty, indicating that procyanidin B2 is remarkably stable under all thermo-sonication conditions tested.

Procyanidin A1 (grand mean = 15.08 ± 0.35 mg/100 mL) demonstrated even greater stability, with an overall CV of 2.3%. The rate constants for the treatments at 35°C, 45°C, and 55°C were k = −0.006 min^−1^ at 35°C (R^2^ = 0.491; +3.06% enhancement), k = 0.011 min^−1^ at 45°C (R^2^ = 0.853; −6.92% degradation), and k = 0.003 min^−1^ at 55°C (R^2^ = 0.187; −2.68% degradation). Treatment of thermo-sonication at 45°C (TS-45°C) had the highest R^2^ (0.853), which indicates a first-order degradation pattern at its intermediate temperature.

Procyanidin A1 was enhanced at TS-35°C (negative k) which suggested an extraction of matrix-bound polyphenols at low-intensity thermo-sonication. The stability of procyanidin during thermo-sonication treatment is a recognized resistance of proanthocyanidin dimers to heat degradation at temperatures beneath 100°C. The procyanidin A1 has a double structure in comparison to procyanidin B2 linkage. The thermo-sonicated samples at 55°C conserved the juices’ procyanidin contents, thus maintaining the bioactive properties essential with this compound, which are the compounds associated in the literature with the anti-adhesion activity of cranberry against uropathogenic *E. coli*, although this bioactivity was not measured experimentally in the present study. The thermal robustness observed here agrees with evidence that the additional C2-O-C7 ether bridge of A-type procyanidins confers greater resistance to thermal and oxidative cleavage than the single interflavan bond of B-type dimers, so that A-type procyanidins are generally well retained during mild processing whereas harsher heat treatments can degrade procyanidin oligomers[49].

#### 3.3.3 Procyanidin temperature sensitivity

Arrhenius analysis was applied to the procyanidin degradation rate constants, with the caveat that only temperatures exhibiting positive rate constants (net degradation) were included. For procyanidin B2, degradation was observed at 35°C (k = 0.006 min^−1^) and 45°C (k = 0.010 min^−1^), with 55°C excluded because it showed net enhancement (k = −0.012 min^−1^). The two-point Arrhenius fit yielded Ea = 20.9 kJ/mol (two-point fit), Q_10_ = 1.29, and z-value = −89.7°C (Table 7). The positive activation energy indicates that B2 degradation follows classical thermally-activated kinetics within the 35-45°C range, where cavitation effects are less dominant than for the aggregate physicochemical parameters. The large magnitude of the z-value (−89.7°C) suggests low temperature sensitivity, constant with thermal resistance of B-type procyanidins at temperatures below 100°C.

**Table 7:** Arrhenius parameters for procyanidin B2 and A1.

**Compound**	**Temperatures Used**	**Ea (kJ/mol)**	**A (min^−1^)**	**R^2^**	**Q_1_**_0_	**z-value (°C)**
**Procyanidin B2**	35, 45°C	20.9	4.34×10^1^	1.000	1.29	−89.7
**Procyanidin A1**	45, 55°C	−53.9	9.41×10^−12^	1.000	0.54	37.1

Degradation was noticed with procyanidin A1 at TS-45°C (k = 0.011 min^−1^) and 55°C (k = 0.003 min^−1^), with TS-35°C excluded from the Arrhenius regression because it showed a net negative rate constant (k = −0.006 min^−1^, i.e., a slight apparent increase rather than degradation), which cannot be represented on an Arrhenius degradation plot. We note that this exclusion is a curve-fitting constraint and does not by itself imply a mechanistic difference at 35°C. The two-point Arrhenius fit yielded Ea = −53.9 kJ/mol (two-point fit), Q_10_ = 0.54, and z-value = 37.1°C (Table 6), consistent with the tabulated parameters.

The negative apparent Ea and low R^2^ are consistent with the acoustic cavitation-dominated degradation mechanisms, and the decreased cavitation intensity results in lower overall rate of degradation. The poor fit quality (R^2^ = 0.291) suggests over-interpretation of the results, that is, the near-zero rate constants and the minor variations with TS-45°C = −6.92%, and TS-55°C= −2.68%, implies the Arrhenius parameters are derivatives from kinetically insignificant variations.

Since each procyanidin fit uses only two temperatures (one rate constant per temperature), these Arrhenius parameters are indicative rather than definitive and should be interpreted with caution; the fit combines the temperature-specific rate constants rather than pooling raw replicates across temperature. The contrasting Arrhenius behaviors of B2 (positive Ea) and A1 (negative Ea) may reflect structural differences in their susceptibility to the competing degradation pathways present during thermo-sonication. The B-type interflavanoid linkage in procyanidin B2 (C4-C8 single bond) is potentially more susceptible to thermal cleavage, consistent with the positive Ea, while the additional C2–O–C7 ether bridge in the A-type procyanidin A1 provides greater structural rigidity and may render it more susceptible to cavitation-mediated radical attack (which decreases with temperature) than to thermal cleavage. Nevertheless, the practical conclusion remains the same for both compounds: thermo-sonication at 35–55°C produces negligible changes in procyanidin content, confirming that this processing technology preserves the characteristic A-type proanthocyanidins that distinguish cranberry from other berry fruits.

### 3.4 Micronutrients: vitamin C, carotenoids, and minerals

#### 3.4.1 Vitamin C

Vitamin C (ascorbic acid) in cranberry juice was significantly influenced by thermo-sonication treatment conditions, though the kinetic patterns varied across the tested temperatures (Table 8). In the US treatment, vitamin C ranged from 1.63 to 2.26 mg/100 mL, with no clear monotonic trend, suggesting that ultrasound-induced sonolysis and oxidation partially counterbalanced with release-enhancement effects.

**Table 8:** Vitamin C and Total Carotenoid concentrations in thermo-sonicated cranberry juice. Different lowercase letters within a column indicate statistically significant differences (Tukey HSD, p < 0.05).

**Treatment**	**Time (min)**	**Vitamin C (mg/100 mL)**	**Total Carotenoids (mg/100 mL)**
US	1	2.26 ± 0.03a	0.82 ± 0.20a
	3	2.22 ± 0.18a	0.94 ± 0.17a
	5	1.63 ± 0.21b	1.75 ± 0.43a
	7	2.16 ± 0.21a	1.89 ± 0.68a
TS-35	1	1.85 ± 0.37a	0.64 ± 0.08b
	3	2.40 ± 0.21a	0.90 ± 0.13a
	5	2.44 ± 0.44a	0.86 ± 0.44a
	7	2.04 ± 0.35a	0.66 ± 0.05b
TS-45	1	2.07 ± 0.25a	0.93 ± 0.06a
	3	1.74 ± 0.22a	1.16 ± 0.05a
	5	1.86 ± 0.05a	1.01 ± 0.03a
	7	1.58 ± 0.08b	0.97 ± 0.20a
TS-55	1	2.02 ± 0.16a	1.57 ± 0.49a
	3	1.69 ± 0.13b	0.93 ± 0.29a
	5	1.71 ± 0.24a	1.61 ± 0.96a
	7	1.72 ± 0.37a	1.01 ± 0.28a

Samples of thermo-sonication at 35 °C and 45 °C (TS-35, and TS-45) had an initial ascorbic acid retention, with moderate decline at higher time points. Thermo-sonicated sample of 35 °C had the highest concentrations of vitamin C of 2.44 mg/100 mL for 5 min (TS 35-5) of treatment, suggesting improved membrane-bond ascorbate pools release during mild thermo-sonication. Thermo-sonicated sample treated at 55 °C gave ascorbic acid values of 1.69 - 2.02 mg/100 mL, with lower values noted for extended treatment durations (3-7 min) compared to 1 min (p < 0.05), consistent with the degradation of ascorbic acids from thermal processing with higher temperatures. The ascorbic acid’s mild losses with thermo-sonication conditions are much lower than the observed losses with traditional thermal pasteurization (70 – 90 °C), suggesting the nutritional advantage of thermo-sonication. The limited ascorbic-acid losses observed here are consistent with reports that thermo-sonication and probe ultrasonication retain vitamin C in apple, strawberry and sweet-lime juices better than conventional thermal pasteurisation, because the shorter effective treatment times and lower bulk temperatures limit oxidative and thermal degradation of ascorbate [48], [17].

#### 3.4.2 Total carotenoids contents

The total carotenoid contents presented a changing kinetic outcome across the samples treated (Table 8). The results of the ultrasound treatment (US) exhibited an increasing total carotenoid contents pattern from 0.82 to 1.89 mg/100 mL for treatments from 1 to 7 min (US-1-7), possibly due to juice matrices structural enhancement of carotenoids extraction from the cavitation disruption.

Thermo-sonication samples at 35°C (TS-35°C) revealed stable carotenoids contents of 0.64–0.90 mg/100 mL, whiles samples of 45°C showed a decrease after an earlier peak at 3 min (TS 35°C – 3 min) with 1.16 mg/100 mL, however, samples of 55°C exhibited higher total catenoids values 1.57 and 1.61 mg/100 mL, respectively at 1 and 5 min of treatment, implying a matrix-bound carotenoids pool release from heat-facilitated extraction. Total carotenoid contents remained low in absolute terms (≤2 mg/100 mL) across all thermo-sonication conditions, consistent with the naturally low carotenoid content of cranberry relative to carotenoid-rich juices; no reference threshold is implied by this comparison The change in carotenoid degradation was not statistically significant within the thermo-sonicated samples. A comparable cavitation-assisted release of carotenoids has been reported for sonicated and thermo-sonicated juices, in which disruption of the cellular matrix increases the extractability of matrix-bound carotenoids [10]; Li et al., 2025).

#### 3.4.3 Mineral profile

The results of mineral composition for the thermo-sonication at 35, 45, 55 °C of cranberry juice samples are presented in Table 9. Macro minerals, calcium (Ca), sodium (Na), potassium (K), and magnesium (Mg), and trace elements zinc (Zn) and iron (Fe) were analyzed. The control cranberry juice sample had Ca - 7.37 mg/L; K - 104.5 mg/L; Na - 3561 mg/L; Mg -5.60 mg/L; Zn - 675 µg/L; and Fe -1377 µg/L. The values of the mineral contents indicate inconsistency across the thermo-sonication treatment durations, underlining effects of the different temperatures on the outcome of the results. Fluctuating mineral profiles were recorded across treatments: TS35-3 and TS45-3 showed higher calcium, sodium and potassium contents than the control (e.g., Ca 10.08 and 8.69 mg/L, K 145.3 and 119.2 mg/L, respectively), which may reflect apparent evaporative concentration during the mild heat treatment. For the ultrasound treatment, Ca and K values moved back towards control levels between 1 and 7 min. These apparent increases and the variability between adjacent time points are unlikely to represent true changes in total mineral content, since sonication and mild heating are not expected to create or destroy mineral elements; instead, they most plausibly arise from treatment-induced differences in sample matrix (e.g., pulp dispersion, partial evaporation, and settling) that affect the fraction of each element detected by ICP-OES rather than the amount present. Interpreted with this caveat, the data indicate that thermo-sonication did not systematically deplete the macro- or micro-minerals of the juice relative to the control, which is consistent with retention of nutritional value under the conditions studied.

**Table 9:** Macro- and micro-mineral profile of cranberry juice subjected to thermo-sonication treatments. Ca, Na, K, Mg reported as mg/L; Zn and Fe reported as µg/L.

**Treatment**	**Ca (mg/L)**	**Na (mg/L)**	**K (mg/L)**	**Mg (mg/L)**	**Zn (µg/L)**	**Fe (µg/L)**
Untreated	7.37 ± 0.00c	3562.00 ± 1.41e	104.50 ± 0.00d	5.50 ± 0.14c	674.50 ± 0.71c	1376.00 ± 1.41c
US-1 min	4.69 ± 0.00l	1715.50 ± 2.12l	81.36 ± 0.04j	3.79 ± 0.00d	402.50 ± 0.71k	817.00 ± 1.41l
US-3 min	5.99 ± 0.00h	2592.00 ± 1.41j	87.03 ± 0.01h	858.00 ± 1.41a	646.50 ± 10.61d	855.00 ± 2.83k
US-5 min	6.30 ± 0.14g	3362.00 ± 1.41g	92.93 ± 0.06f	4.83 ± 0.00c	668.00 ± 1.41c	1297.00 ± 1.41d
US-7 min	7.06 ± 0.00d	3854.50 ± 0.71d	102.35 ± 0.07e	4.01 ± 0.00d	665.50 ± 2.12c	1052.50 ± 0.71g
TS35-1 min	5.32 ± 0.00k	1061.00 ± 1.41p	80.68 ± 0.01j	4.05 ± 0.00d	498.00 ± 1.41g	765.00 ± 1.41n
TS35-3 min	10.07 ± 0.01a	5714.00 ± 1.41b	145.50 ± 0.28a	7.64 ± 0.00b	878.00 ± 1.41a	1463.00 ± 1.41a
TS35-5 min	3.80 ± 0.00n	0.09 ± 0.00q	66.38 ± 0.01n	3.02 ± 0.00d	322.00 ± 1.41m	537.00 ± 1.41p
TS35-7 min	5.84 ± 0.00j	1438.00 ± 1.4n1	88.37 ± 0.01g	4.43 ± 0.00c	548.00 ± 1.41f	888.00 ± 1.41j
TS45-1 min	3.78 ± 0.00n	1201.00 ± 1.41o	61.37 ± 0.01o	2.96 ± 0.00d	437.00 ± 1.41j	418.00 ± 1.41q
TS45-3 min	8.69 ± 0.00b	5218.00 ± 1.41c	119.30 ± 0.14c	7.05 ± 0.00b	802.00 ± 1.41b	1198.00 ± 1.41e
TS45-5 min	4.64 ± 0.00l	1646.00 ± 1.41m	77.73 ± 0.01m	3.73 ± 0.00d	413.00 ± 1.41k	947.00 ± 1.41j
TS45-7 min	4.25 ± 0.00m	5837.00 ± 1.41a	72.54 ± 0.07a	3.42 ± 0.00d	369.00 ± 0.00l	608.00 ± 1.41o
TS55-1 min	5.47 ± 0.00j	3457.00 ± 1.41f	74.53 ± 0.03f	3.69 ± 0.00d	453.00 ± 1.41j	1015.00 ± 1.41h
TS55-3 min	6.46 ± 0.00f	1758.00 ± 1.41k	93.08 ± 0.02k	4.84 ± 0.00c	575.00 ± 2.83e	1165.00 ± 1.41f
TS55-5 min	5.85 ± 0.00h	2795.00 ± 1.41h	102.60 ± 0.14h	4.73 ± 0.01c	481.00 ± 1.41h	797.00 ± 1.41m
TS55-7 min	6.80 ± 0.00e	2162.00 ± 1.41j	108.30 ± 0.28j	5.48 ± 0.00c	651.00 ± 1.41d	1385.00 ± 1.41b

### 3.5 Color parameters, pH, and total soluble solids

The effects of thermo-sonication at 35, 45 and 55°C on color parameters, pH and total solids are presented in Table 10.

**Table 10:** CIE L*a*b* color coordinates, total color change (ΔE), pH, and total soluble solids (TSS, °Brix) of thermo-sonicated cranberry juice

**Treatment**	**TSS (°Brix)**	**pH**	L*	a*	b*	**ΔE**
Untreated	9.80 ± 0.73 a	2.60 ± 0.16 a	21.15 ± 0.07 c	10.46 ± 0.06 g	5.25 ± 0.18 e	-
US-1 min	9.97 ± 0.78 a	2.53 ± 0.18 a	19.82 ± 0.21 f	10.96 ± 0.11 f	5.75 ± 0.02 d	1.51 ± 0.14 g
US-3 min	9.75 ± 0.97 a	2.48 ± 0.14 a	19.46 ± 0.04 f	11.14 ± 0.01 f	5.12 ± 0.01 e	1.82 ± 0.04 f
US-5 min	9.65 ± 1.17 a	2.51 ± 0.14 a	20.31 ± 0.01 e	10.73 ± 0.02 g	5.32 ± 0.02 e	0.88 ± 0.01 i
US-7 min	9.78 ± 1.18 a	2.50 ± 0.17 a	20.66 ± 0.04 d	11.55 ± 0.01 e	4.74 ± 0.04 f	1.29 ± 0.01 g
TS35-1 min	9.62 ± 0.68 a	2.50 ± 0.15 a	21.07 ± 0.05 d	12.94 ± 0.08 c	6.13 ± 0.01 c	2.63 ± 0.07 e
TS35-3 min	9.53 ± 0.88 a	2.52 ± 0.16 a	20.44 ± 0.03 e	11.71 ± 0.08 e	5.38 ± 0.05 e	1.44 ± 0.08 g
TS35-5 min	8.88 ± 0.78 a	2.51 ± 0.16 a	21.58 ± 0.09 c	15.33 ± 0.16 b	7.53 ± 0.07 a	5.39 ± 0.16 c
TS35-7 min	9.57 ± 0.39 a	2.53 ± 0.15 a	21.51 ± 0.04 c	13.03 ± 0.01 c	5.46 ± 0.04 d	2.60 ± 0.02 e
TS45-1 min	7.25 ± 1.07 b	2.57 ± 0.20 a	21.59 ± 0.21 c	15.04 ± 0.01 b	6.88 ± 0.10 b	4.88 ± 0.06 d
TS45-3 min	9.82 ± 0.96 a	2.50 ± 0.14 a	21.27 ± 0.06 c	12.81 ± 0.10 c	5.48 ± 0.04 d	2.36 ± 0.11 e
TS45-5 min	9.62 ± 0.05 a	2.52 ± 0.13 a	26.39 ± 0.09 a	9.13 ± 0.01 h	3.19 ± 0.05 g	5.79 ± 0.10 b
TS45-7 min	8.45 ± 0.37 a	2.53 ± 0.16 a	23.15 ± 0.06 b	17.54 ± 0.03 a	7.56 ± 0.17 a	7.71 ± 0.09 a
TS55-1 min	9.07 ± 0.21 a	2.52 ± 0.14 a	20.61 ± 0.23 e	11.44 ± 0.14 e	5.63 ± 0.04 d	1.20 ± 0.00 h
TS55-3 min	6.80 ± 1.27 b	2.56 ± 0.18 a	19.69 ± 0.06 f	11.42 ± 0.01 e	6.05 ± 0.04 c	1.92 ± 0.02 f
TS55-5 min	7.15 ± 1.17 b	2.57 ± 0.18 a	20.88 ± 0.01 d	12.35 ± 0.03 d	6.03 ± 0.01 c	2.06 ± 0.03 f
TS55-7 min	7.65 ± 0.97 b	2.55 ± 0.17 a	21.22 ± 0.16 c	12.38 ± 0.06 d	5.23 ± 0.02 e	1.91 ± 0.06 f

Different lowercase letters within a column indicate statistically significant differences (Tukey HSD, p < 0.05). TSS and pH are means of four replicates (n = 4); L*, a*, b* and ΔE are means of duplicate readings (n = 2); ΔE is relative to the untreated control mean

#### 3.5.1 Color parameters (L*, a*, b*, ΔE)

The color parameter values of the control untreated cranberry juice sample were of L* - 21.20 (lightness), a* -10.51 (redness), and b* -5.37 (yellowness). The ultrasonicated treatment produced modest color changes, with ΔE values ranging from 1.12 to 1.76 across the treatment times 1–7 min, changes that would be perceptible but minor to a trained observer (Table 10). In contrast, thermo-sonication produced more pronounced and temperature-dependent color modifications.

TS-35°C increased redness (a*) substantially, particularly at 5 min (a* - 15.44), corresponding to ΔE - 5.10, the largest single-point color change among all US treatments. This enhanced redness may reflect the conversion of colorless procyanidin forms or flavonoids to colored anthocyanin-related species under mild thermal and acoustic conditions, or alternatively the concentration of chromophoric compounds through minor water loss. TS-45°C produced the most variable and overall highest color changes, with a* reaching 17.56 and ΔE peaking at 7.23 at 7 min, associated with an increase in L* at 5 min (L* = 26.46) suggesting lightness changes possibly from anthocyanin structural rearrangement or co-precipitation of coloring matter.

TS-55°C produced relatively moderate color changes (ΔE - 1.11-1.93), with redness remaining below 12.42 throughout treatment, suggesting that higher temperatures lead to partial decomposition of red anthocyanin pigments, reducing overall redness. This is constant with the decreased total anthocyanin contents (TAC) noted with TS-55°C against TS-45°C corresponding treatment time points. Overall, the results of the color data suggest that moderate temperature, that is between 35°C to 45°C with sonication improves red color characteristics of cranberry juice, whereas ultrasonication alone mildly changed color, thus mild thermo-sonication would be highly recommended for processing where color preservation is required. The moderate, temperature-dependent colour changes and the enhancement of redness at 35-45 °C agree with reports that mild thermo-sonication can brighten and intensify juice colour through cavitation-assisted pigment release, whereas higher temperatures promote anthocyanin decomposition and a loss of redness (Li et al., 2025; [17].

#### 3.5.2 pH and total soluble solids (TSS)

The untreated control cranberry juice had a pH of 2.60 (Table 10). Only minor changes in pH were observed after treatment, with values remaining within a narrow range (approximately 2.48–2.60) across all ultrasound and thermo-sonication conditions, and no statistically significant differences among treatments (Tukey HSD, p > 0.05). The minor pH alterations (< 0.12 units) could have effects on the cranberry juice’s sensory characteristics, or stability, since a key characteristic quality of cranberry juice is its low pH which contributes to its antibacterial properties and stable shelf life. Overall, the total soluble solids (TSS) decreased from 10.9 brix for the control to 7.7-9.7 brix observed with the treated samples, which could be because of the fructose dilution caused by acoustic cavitation and Maillard reaction during the thermo-sonication treatment, as reported in this other studies referenced (L. Li et al., 2025; [50], [51]. Total soluble solids fluctuated modestly across treatments (approximately 6.8-9.8 °Brix; Table 10), with no statistically significant differences among treatments in the present data (Tukey HSD, p > 0.05). Any small variations are of limited practical significance from a formulation perspective.

#### 3.5.3 Microbial analysis

The effect of ultrasonication at 25°C on the total plate count (TPC) of cranberry juice is presented on Fig. 8(a). The untreated control recorded a TPC of 5.602 log CFU/mL. The ultrasonicated cranberry juice samples produced statistically significant decreases in microbial counts analysis relative to the control (at p < 0.05). The ultrasound treated cranberry sample for 1 min (US-1) had a reduction of 0.951 log CFU/mL at 4.651 ± 0.069 log CFU/mL count, this corresponds to 89% decrease in viable microbial counts. Effective microbial inactivation was also observed from the 3 min (US-3) treatment onwards, with samples 3, 5, and 7 min (US - 3,5 and 7), producing identical values of 3.602 ± 0.000 log CFU/mL count, representing a 2.000 log reduction hence 99% inactivation in comparison to the control.

**Fig. 8:**
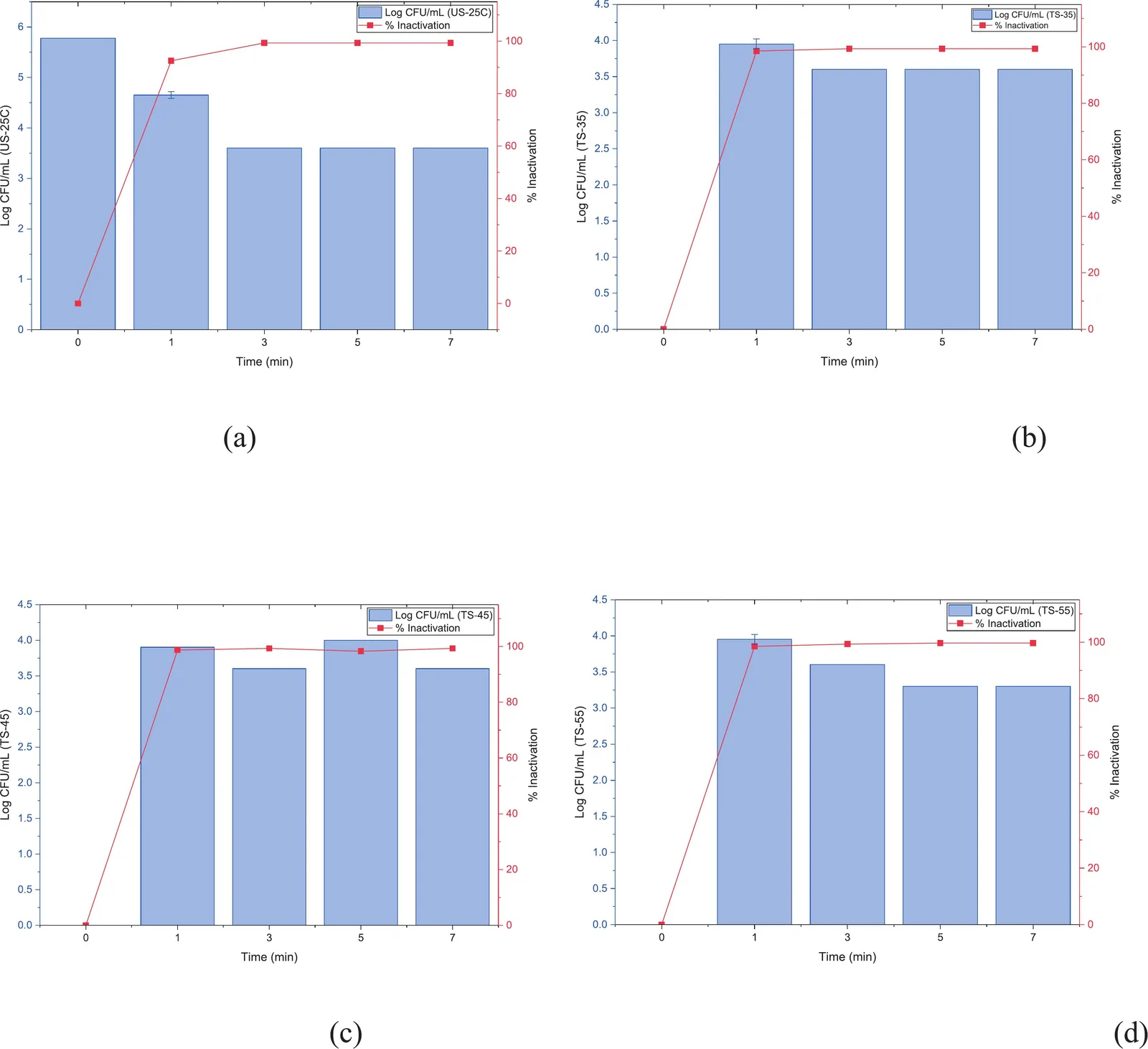
Microbial inactivation analysis of thermo-sonicated cranberry juice at different temperatures (a) room temperature 25°C; (b) 35°C; (c) 45 ^o^C; and (d) 55°C.

The effect of the thermo-sonication treatment of cranberry juice at 35°C on the total plate count of cranberry juice is shown on Fig. 8(b). The control untreated sample had a total plate count of 5.778 log CFU/mL. TS-35°C - 1 min diminished the total plate count to 3.952 ± 0.069 log CFU/mL (98.5% deactivation). The total plate count reached a plateau of 3.602 ± 0.000 log CFU/mL, representing a 99.3% inactivation, with 3, 5, and 7 min of combined treatments, in each case (p < 0.05). The maximum achievement of inactivation at 3 min, with TS-35°C -3 min, implies that the acoustic cavitation and mild heat, accelerated microbes’ membrane disruption, causing a deactivation threshold to be achieved with a minimal treatment time of 3 min.

The antimicrobial mechanisms involved in the thermo-sonication process at near-ambient temperatures (35°C) involves a synergistic interaction between thermal stress and acoustic cavitation. Although 35°C is sub-lethal for most mesophilic organisms in isolation, concurrent sonication generates localised thermal hot spots at cavitation collapse sites that substantially exceed the bulk temperature, causing membrane fluidity changes that facilitate RONS-mediated oxidative damage and mechanical lysis. The consistent 2.176 log reductions at T35-3 through T35-7 confirm that 3 min at 35°C is the minimum effective treatment duration for maximum thermo-sonication inactivation under the tested experimental conditions.

The microbial inactivation profile of thermo-sonication at 45°C is presented on Fig. 8(c). The untreated control recorded a TPC of 5.778 log CFU/mL. TS-45°C -1min, had a total plate count decrease of 3.903 ± 0.000 log CFU/mL; TS-45°C – 3min achieved 3.602 ± 0.000 log CFU/mL; TS-45°C - 5min resulted in 4.000 ± 0.000 log CFU/mL, while TS-45 °C - 7 min maintained a comparable reduction; all reductions were statistically significant relative to the untreated control (p < 0.05). The fluctuating patterns observed at TS-45-5 min, having a higher total plate count (TPC) than TS-45-3, and TS-45-7 may suggest a thermotolerant sub-group that is resistant to combined treatment with mild heat and ultrasound stress at intermediate treatment times.

The effect of thermo-sonication at 55°C on the total plate count (TPC) of cranberry juice is presented on Fig. 8(d). The control untreated sample had a total plate count (TPC) of 5.778 log CFU/mL. Thermo-sonication treatment at 55°C resulted in a dose-response relationship with treatment times. TS-55°C-1min decreased the TPC to 3.952 ± 0.069 log CFU/mL (98,5%) deactivation, TS-55°C -3min further decreased it to 3.602 ± 0.000 log CFU/mL, a 99.3% deactivation, while TS-55 °C – 5 min and TS-55 °C – 7 min both achieved 3.301 ± 0.000 log CFU/mL, corresponding to a 2.477 log reduction (99.7% inactivation). Thermo-sonication of 55 °C resulted in the most efficient approach among all the treatments. The 2.0-2.5 log reductions achieved here are consistent with, though somewhat lower than, the reductions reported for thermo-sonicated juices at comparable or higher temperatures (for example, reductions exceeding 5-6 log in kiwi and blueberry juices at 55 °C), differences that reflect the initial microbial load, juice matrix and applied acoustic energy; as in those studies, combining mild heat with ultrasound achieved microbial inactivation faster and at a lower temperature than mild heat alone[48], [16].

## 4 Conclusion

This study characterised the kinetics of the effect of thermo-sonication (35, 45 and 55 °C; 1-7 min) on the physicochemical properties, micronutrients, key bioactive compounds and colour of cranberry juice, benchmarked against ultrasonication alone. Ultrasound treatment preserved and, at 3 min, transiently enhanced the total phenolic content (TPC; peak of 12.85 mg GAE/100 mL at US-3) through a biphasic extraction-degradation mechanism, whereas thermo-sonication generally lowered TPC. Thermo-sonication at 35 °C uniquely produced a progressive, nearly three-fold increase in FRAP antioxidant capacity (0.64 to 1.78 mmol Fe^2+^/L over 7 min), indicating improved bioaccessibility of reducing compounds. Most importantly for cranberry, the characteristic A-type and B-type procyanidins (A1 and B2) remained statistically unchanged under all conditions (p > 0.05), confirming that thermo-sonication preserves the hallmark bioactive compounds that underpin cranberry's anti-adhesion functionality. Anthocyanins decreased with all treatments, but the thermo-sonicated samples retained more anthocyanin than ultrasound alone at 7 min (up to 130.70 mg CGE/L at 35 °C), and 45 °C gave the highest redness (a* up to 17.56) while pH remained stable. Vitamin C, carotenoids and the mineral profile were largely conserved. Microbiologically, thermo-sonication achieved 2.0-2.5 log reductions, with the greatest inactivation at 55 °C, reaching the effective threshold within 3 min. Overall, thermo-sonication at 35 °C offered the best combination of antioxidant enhancement and procyanidin preservation, while 45 °C best preserved anthocyanins and colour. Future work would focus on shelf-life and sensory evaluation to support industrial adoption of thermo-sonication for cranberry juice processing.

## CRediT authorship contribution statement

**Mabel Kyei Kwofie:** Writing – original draft, Validation, Methodology, Investigation, Formal analysis, Conceptualization. **Valerie Orsat:** Writing – review & editing, Validation, Supervision, Investigation, Funding acquisition.

## Declaration of competing interest

The authors declare that they have no known competing financial interests or personal relationships that could have appeared to influence the work reported in this paper.

## Appendix A Supplementary data

Supplementary Data 1 Supplementary data of Thermosonication Kinetics of Cranberry Juice.
