# Supplementary material for: Effect of thermo-sonication on the physicochemical properties, bioactive compounds, micronutrients, and antioxidant activity of cranberry juice
Source: Ultrason Sonochem. 2026 Oct 6;134:108098. doi: 10.1016/j.ultsonch.2026.108098 (PMC13675634; doi:10.1016/j.ultsonch.2026.108098)
Supplement: Supplementary file 1 [file mmc1.docx]

**Supplementary Material**

*Effect of Thermo-sonication on the Physicochemical Properties, Bioactive Compounds, Micronutrients, and Antioxidant Activity of Cranberry Juice*

*M. K. Kwofie and V. Orsat — Bioresource Engineering Department, McGill University*

**S1. LC–MS analytical validation**

Procyanidin A1 and procyanidin B2 were identified and quantified by liquid chromatography coupled to electrospray-ionization quadrupole time-of-flight tandem mass spectrometry (LC-ESI-Q-TOF-MS/MS) operated in positive-ion mode with a reversed-phase C18 column. Compounds were confirmed from their accurate-mass precursor ions and retention times against authentic standards and quantified against external calibration curves of those standards. The analytical parameters are summarised in Table S1; representative extracted-ion chromatograms (EIC) and the corresponding mass spectra of both compounds in the standards and in a treated sample are shown in Figure S1.

**Table S1. LC–ESI–Q–TOF–MS/MS identification and calibration parameters for procyanidin A1 and B2.**

| **Compound** | **Molecular formula** | **m/z ([M–H]⁻)** | **Retention time (min)** | **Calibration range (ng/mL)** | **LOD (ng/mL)** | **LOQ (ng/mL)** |
| --- | --- | --- | --- | --- | --- | --- |
| Procyanidin A1 | C₃₀H₂₄O₁₂ | 577.1341 | 3.90 | 10–2000 | 4.88 | 16.3 |
| Procyanidin B2 | C₃₀H₂₆O₁₂ | 579.1497 | 3.60 | 10–500 | 2.42 | 8.1 |

**Figure S1. (A) Extracted-ion chromatograms (EIC) and (B) corresponding mass spectra of procyanidin A1 and B2 in the standard and in a thermo-sonicated cranberry juice sample. A1 (C₃₀H₂₄O₁₂) elutes at ~3.90 min (m/z 577.1341) and B2 (C₃₀H₂₆O₁₂) at ~3.60 min (m/z 579.1497).**

**S2. Kinetic modelling: models, fitting and model-comparison criteria**

For each measured parameter (TFC, TPC, FRAP, DPPH and TAC) and each treatment (US-25, TS-35, TS-45, TS-55), three candidate models were fitted to the replicate-level data (three replicates at each of the treatment times t = 0, 1, 3, 5 and 7 min; n = 15 observations per series, except where a time point was unavailable) by non-linear least squares (Levenberg–Marquardt; scipy.optimize.curve_fit):

• First-order (single exponential): C(t) = C₀ · exp(−k·t)	- 2 parameters.

• Biphasic (bi-exponential): C(t) = A · exp(−k₁·t) + B · exp(−k₂·t) - 4 parameters. This form can describe a fast phase followed by a slow phase and, with opposite-signed amplitudes, a non-monotonic (rise-then-fall) profile.

• Weibull: C(t) = C₀ · exp[−(t/α)^β] -3 parameters; α is the scale (time) and β the shape parameter.

For every fit we report the parameter estimates with 95% confidence intervals (CI = estimate ± t₀.₉₇₅,df · SE, SE from the covariance matrix), the coefficient of determination (R²) and adjusted R², and the root-mean-square error (RMSE). Residual behaviour was assessed with the Shapiro-Wilk test for normality (p > 0.05 indicates no departure from normality) and the Durbin-Watson (DW) statistic for first-order serial correlation (DW ≈ 2 indicates no autocorrelation). Models were compared with the Akaike Information Criterion, AIC = n·ln(RSS/n) + 2K, and the small-sample-corrected AICc = AIC + 2K(K+1)/(n−K−1), where K is the number of fitted parameters. For each series the preferred model is the one with the lowest AIC; ΔAIC = AIC − AIC_min and Akaike weights (wᵢ = exp(−ΔAICᵢ/2) / Σ exp(−ΔAICⱼ/2)) quantify the relative support for each model. Because several series are clearly non-monotonic, the first-order model is used in the main text as an empirical descriptor of the overall rate of change rather than as a mechanistic model; the analysis below makes the quality of each fit explicit.

**S3. First-order regression equations, rate constants and 95% confidence intervals**

Rate constants were estimated by regressing ln(C/C₀) against time. A positive k denotes net degradation, and a negative k denotes net enhancement (accumulation/extraction). Derived indices: half-life t½ = ln2/k and decimal reduction time D = ln10/k (reported only where k > 0).

**Table S2. First-order regression equations, rate constants k with 95% CI, goodness-of-fit and half-life.**

| **Parameter** | **Treatment** | **Regression equation** | **k (min⁻¹)** | **95% CI of k** | **R²** | **RMSE** | **t½ (min)** |
| --- | --- | --- | --- | --- | --- | --- | --- |
| TFC | US-25 | C(t) = 6.61·exp(−(-0.0117)·t) | -0.0117 | [-0.0778, 0.0544] | 0.012 | 1.949 | — |
| TFC | TS-35 | C(t) = 5.73·exp(−(-0.0177)·t) | -0.0177 | [-0.0737, 0.0383] | 0.032 | 1.465 | — |
| TFC | TS-45 | C(t) = 7.34·exp(−(-0.0093)·t) | -0.0093 | [-0.0827, 0.0641] | 0.005 | 2.382 | — |
| TFC | TS-55 | C(t) = 7.01·exp(−(0.0558)·t) | 0.0558 | [0.0147, 0.0970] | 0.413 | 0.994 | 12.41 |
| TPC | US-25 | C(t) = 8.00·exp(−(-0.0576)·t) | -0.0576 | [-0.1207, 0.0055] | 0.242 | 2.648 | — |
| TPC | TS-35 | C(t) = 6.56·exp(−(0.1132)·t) | 0.1132 | [0.0235, 0.2028] | 0.400 | 1.602 | 6.12 |
| TPC | TS-45 | C(t) = 6.56·exp(−(0.1067)·t) | 0.1067 | [0.0129, 0.2005] | 0.351 | 1.721 | 6.50 |
| TPC | TS-55 | C(t) = 6.18·exp(−(0.0951)·t) | 0.0951 | [0.0135, 0.1767] | 0.335 | 1.480 | 7.29 |
| FRAP | US-25 | C(t) = 0.49·exp(−(0.1290)·t) | 0.1290 | [0.0742, 0.1837] | 0.688 | 0.068 | 5.37 |
| FRAP | TS-35 | C(t) = 0.78·exp(−(-0.1220)·t) | -0.1220 | [-0.1445, -0.0994] | 0.923 | 0.114 | — |
| FRAP | TS-45 | C(t) = 0.49·exp(−(0.0682)·t) | 0.0682 | [0.0276, 0.1089] | 0.514 | 0.066 | 10.16 |
| FRAP | TS-55 | C(t) = 0.42·exp(−(0.0807)·t) | 0.0807 | [0.0011, 0.1602] | 0.251 | 0.105 | 8.59 |
| DPPH | US-25 | C(t) = 22.39·exp(−(0.0281)·t) | 0.0281 | [-0.0608, 0.1170] | 0.029 | 7.644 | 24.67 |
| DPPH | TS-35 | C(t) = 26.59·exp(−(0.0788)·t) | 0.0788 | [0.0079, 0.1497] | 0.321 | 5.912 | 8.79 |
| DPPH | TS-45 | C(t) = 26.55·exp(−(0.1734)·t) | 0.1734 | [-0.0055, 0.3523] | 0.298 | 10.047 | 4.00 |
| DPPH | TS-55 | C(t) = 24.10·exp(−(0.1145)·t) | 0.1145 | [-0.0167, 0.2457] | 0.232 | 8.561 | 6.05 |
| TAC | US-25 | C(t) = 75.74·exp(−(0.1815)·t) | 0.1815 | [-0.0554, 0.4184] | 0.259 | 36.690 | 3.82 |
| TAC | TS-35 | C(t) = 61.92·exp(−(-0.0982)·t) | -0.0982 | [-0.2113, 0.0150] | 0.207 | 42.032 | — |
| TAC | TS-45 | C(t) = 57.26·exp(−(-0.0827)·t) | -0.0827 | [-0.1836, 0.0183] | 0.188 | 32.989 | — |
| TAC | TS-55 | C(t) = 39.51·exp(−(-0.1166)·t) | -0.1166 | [-0.3018, 0.0687] | 0.091 | 46.530 | — |

**S4. Residual diagnostics (first-order fits)**

**Table S3. Residual diagnostics for the first-order fits: adjusted R², Shapiro–Wilk normality p, Durbin–Watson (DW), and flags.**

| **Parameter** | **Treatment** | **Adj. R²** | **Shapiro–Wilk p** | **DW** | **Diagnostic flags** |
| --- | --- | --- | --- | --- | --- |
| TFC | US-25 | -0.153 | 0.295 | 0.96 | autocorrelation; poor fit (low R²) |
| TFC | TS-35 | -0.129 | 0.014 | 1.08 | non-normal residuals; autocorrelation; poor fit (low R²) |
| TFC | TS-45 | -0.160 | 0.018 | 1.31 | non-normal residuals; autocorrelation; poor fit (low R²) |
| TFC | TS-55 | 0.315 | 0.996 | 2.21 | OK |
| TPC | US-25 | 0.115 | 0.131 | 1.09 | autocorrelation; poor fit (low R²) |
| TPC | TS-35 | 0.300 | 0.049 | 1.06 | non-normal residuals; autocorrelation |
| TPC | TS-45 | 0.243 | 0.304 | 1.44 | autocorrelation |
| TPC | TS-55 | 0.224 | 0.073 | 0.99 | autocorrelation |
| FRAP | US-25 | 0.636 | 0.539 | 0.90 | autocorrelation |
| FRAP | TS-35 | 0.910 | 0.865 | 1.29 | autocorrelation |
| FRAP | TS-45 | 0.433 | 0.939 | 1.13 | autocorrelation |
| FRAP | TS-55 | 0.126 | 0.543 | 0.68 | autocorrelation; poor fit (low R²) |
| DPPH | US-25 | -0.133 | 0.520 | 1.15 | autocorrelation; poor fit (low R²) |
| DPPH | TS-35 | 0.208 | 0.493 | 1.67 | OK |
| DPPH | TS-45 | 0.181 | 0.095 | 1.58 | poor fit (low R²) |
| DPPH | TS-55 | 0.104 | 0.532 | 1.49 | autocorrelation; poor fit (low R²) |
| TAC | US-25 | 0.136 | 0.006 | 1.02 | non-normal residuals; autocorrelation; poor fit (low R²) |
| TAC | TS-35 | 0.075 | 0.327 | 1.06 | autocorrelation; poor fit (low R²) |
| TAC | TS-45 | 0.053 | 0.269 | 1.06 | autocorrelation; poor fit (low R²) |
| TAC | TS-55 | -0.060 | 0.259 | 1.65 | poor fit (low R²) |

**Figure S3. Representative residual diagnostics for a well-fitted series (FRAP, TS-35): residuals versus fitted values (left) and normal Q–Q plot (right).**

**S5. Formal model comparison (AIC) against biphasic and Weibull models**

**Table S4. Model comparison by AIC. Each cell gives AIC (Akaike weight wᵢ); lower AIC and higher wᵢ indicate stronger support. The last column is the preferred model.**

| **Parameter** | **Treatment** | **First-order AIC (w)** | **Biphasic AIC (w)** | **Weibull AIC (w)** | **Preferred model** |
| --- | --- | --- | --- | --- | --- |
| TFC | US-25 | 24.0 (0.65) | 28.0 (0.09) | 25.8 (0.26) | First-order |
| TFC | TS-35 | 15.5 (0.29) | 13.9 (0.63) | 17.9 (0.08) | Biphasic |
| TFC | TS-45 | 30.0 (0.48) | 30.6 (0.35) | 32.1 (0.17) | First-order |
| TFC | TS-55 | 3.8 (0.65) | 7.8 (0.09) | 5.7 (0.26) | First-order |
| TPC | US-25 | 33.2 (0.85) | 37.2 (0.11) | 39.4 (0.04) | First-order |
| TPC | TS-35 | 18.1 (0.06) | 14.7 (0.35) | 13.7 (0.59) | Weibull |
| TPC | TS-45 | 20.3 (0.12) | 18.3 (0.31) | 17.1 (0.57) | Weibull |
| TPC | TS-55 | 15.8 (0.00) | 0.2 (0.56) | 0.7 (0.44) | Biphasic |
| FRAP | US-25 | -76.8 (0.00) | -96.0 (0.28) | -97.9 (0.72) | Weibull |
| FRAP | TS-35 | -61.1 (0.88) | -57.1 (0.12) | -20.7 (0.00) | First-order |
| FRAP | TS-45 | -77.6 (0.01) | -85.1 (0.45) | -85.5 (0.53) | Weibull |
| FRAP | TS-55 | -63.6 (0.00) | -97.1 (1.00) | -86.0 (0.00) | Biphasic |
| DPPH | US-25 | 65.0 (0.00) | 46.6 (0.99) | 56.9 (0.01) | Biphasic |
| DPPH | TS-35 | 57.3 (0.03) | 52.0 (0.37) | 51.0 (0.61) | Weibull |
| DPPH | TS-45 | 73.2 (0.29) | 73.8 (0.21) | 72.1 (0.50) | Weibull |
| DPPH | TS-55 | 68.4 (0.03) | 63.1 (0.37) | 62.1 (0.60) | Weibull |
| TAC | US-25 | 112.1 (0.33) | 116.1 (0.05) | 110.8 (0.62) | Weibull |
| TAC | TS-35 | 116.2 (0.79) | 119.3 (0.16) | 121.6 (0.05) | First-order |
| TAC | TS-45 | 108.9 (0.79) | 112.2 (0.15) | 114.0 (0.06) | First-order |
| TAC | TS-55 | 119.2 (0.04) | 112.8 (0.95) | 122.6 (0.01) | Biphasic |

**Figure S2. Representative fits of the three candidate models to the replicate-level data for TPC (TS-35), FRAP (TS-35), DPPH (TS-45) and TAC (TS-45). AIC values are given in the legends.**

**S6. Interpretation and justification of the first-order treatment**

The diagnostics show that several series are not well described by a single-exponential model. For the FRAP response, which is monotonic, the first-order model is strongly supported (e.g., TS-35: R² ≈ 0.92, normally distributed residuals, and the lowest AIC among the three models). For TFC, TPC, DPPH and TAC the first-order R² values are modest (typically 0.0-0.4) and, for the non-monotonic series, the biphasic or Weibull model achieves a lower AIC; however, the differences in AIC are generally small and the added parameters are not always justified given n = 15 and the pronounced replicate variability. We therefore (i) retain the first-order model in the main text only as an empirical descriptor of the overall rate and direction of change, (ii) report R² for every fit and explicitly flag poor fits, and (iii) avoid over-interpreting rate constants derived from low-R² or clearly biphasic series. Where a biphasic or Weibull model is preferred by AIC, this is noted as evidence of competing extraction and degradation processes operating on different time scales rather than a single first-order decay.
